# Reducing Sanger confirmation testing through false positive prediction algorithms

**DOI:** 10.1038/s41436-021-01148-3

**Published:** 2021-03-25

**Authors:** James M. Holt, Melissa Kelly, Brett Sundlof, Ghunwa Nakouzi, David Bick, Elaine Lyon

**Affiliations:** HudsonAlpha Institute for Biotechnology, Huntsville, AL USA

## Abstract

**Purpose:**

Clinical genome sequencing (cGS) followed by orthogonal confirmatory testing is standard practice. While orthogonal testing significantly improves specificity, it also results in increased turnaround time and cost of testing. The purpose of this study is to evaluate machine learning models trained to identify false positive variants in cGS data to reduce the need for orthogonal testing.

**Methods:**

We sequenced five reference human genome samples characterized by the Genome in a Bottle Consortium (GIAB) and compared the results with an established set of variants for each genome referred to as a truth set. We then trained machine learning models to identify variants that were labeled as false positives.

**Results:**

After training, the models identified 99.5% of the false positive heterozygous single-nucleotide variants (SNVs) and heterozygous insertions/deletions variants (indels) while reducing confirmatory testing of nonactionable, nonprimary SNVs by 85% and indels by 75%. Employing the algorithm in clinical practice reduced overall orthogonal testing using dideoxynucleotide (Sanger) sequencing by 71%.

**Conclusion:**

Our results indicate that a low false positive call rate can be maintained while significantly reducing the need for confirmatory testing. The framework that generated our models and results is publicly available at https://github.com/HudsonAlpha/STEVE.

## INTRODUCTION

Clinical next-generation sequencing (NGS) is widely used to identify a molecular diagnosis in patients with suspected genetic disorders.^1,2^ Unfortunately, NGS pipelines are known to have both random and systematic errors at sequencing, alignment, and variant calling steps of the pipeline.^3,4^ Because the reported variants can impact patient care, the American College of Medical Genetics and Genomics (ACMG) and the College of American Pathologists (CAP) recommend orthogonal confirmation (e.g., Sanger sequencing) for reported variants to reduce the risk of false positive results.^3,4^ Unfortunately, orthogonal confirmation increases both the cost and turnaround time of the NGS test. Furthermore, the total number of variants that are candidates for clinical reporting is steadily increasing, as demonstrated by the growth in public databases such as ClinVar and OMIM.^5,6^ Orthogonal confirmation of all reported variants will cause the effective cost of NGS to steadily increase due to an increase in the number of variants sent for confirmation.

To address this issue, other studies have questioned the necessity of orthogonal testing, especially when the variant call is of sufficiently high quality for the particular NGS assay.^7–10^ Most of these studies involved a relatively small sample size (<8,000 variants), with the notable exception of the work by Lincoln et al., which examined approximately 200,000 variants, identifying 1,662 as false positives.^10^ Lincoln et al. used a combination of reference samples characterized by the Genome in a Bottle Consortium (GIAB)^11–13^ along with orthogonal test results from over 80,000 clinical tests from two different laboratories. Briefly, their method involved manual selection of candidate thresholds for quantitative metrics that were then converted into flags. Then, these flags were provided as input to a heuristic algorithm to classify variant calls as high-confidence true positive calls or candidate true positive calls (requiring confirmation testing). The exact set of flags was notably different for single-nucleotide variants (SNVs) and insertions/deletions (indels). The authors establish a 100% capture rate (lower bound on confidence interval 98.5–98.9% for SNVs, 99.1–99.8% for indels) for false positive calls while maintaining relatively low rates of true calls that were incorrectly flagged using their approach (4.1–13.2% for SNVs, 6.7–15.4% for indels).^10^

Despite the success of the Lincoln et al. approach, there are some drawbacks that make broad application challenging. First, their data were gathered from custom hybridization-based assays, limiting the scope to targeted regions of the genome. This led to relatively few false positive variant calls (1,662 of 200,000 variants) across their entire data set, and likely contributed to the wide confidence intervals for some of the false positive capture rates (SNVs in particular). This also limited the variants they could use from GIAB truth sets requiring the study to rely on a relatively large number of orthogonally confirmed results performed by the laboratory as part of their clinical testing (>80,000). For many labs, this is impractical due to costs, especially when developing a new test where orthogonal results are not already known. Second, the selection of flags from quality metrics is a manual step in their process that also reduces the information content of the metrics. Reduction to flag values (e.g., a Boolean flag value) due to the use of discrete thresholds can reduce the ability of the algorithm to detect complex interactions between quality metrics, especially when those interactions are based on multiple different thresholds/ranges across multiple different quality metrics. While multiple thresholds could be used (as Lincoln et al. did), this still results in a loss of information compared with the unaltered quantitative metric.

To address difficulties associated with Lincoln’s approach, we applied an automated machine learning approach^14,15^ that uses the entirety of the GIAB truth sets as the training and testing sets as is fitting for our application of clinical genome sequencing (cGS). The benefits of this approach are threefold: (1) automation of quality metric evaluation on non-Boolean values (i.e., no manually identified flags), (2) a substantial increase in the number of true positive and false positive variant calls available for training and testing (~3.2–3.5 million true positives per sample) due to our use of cGS, and (3) elimination of orthogonal testing results in algorithm training due to the abundance of data from the clinical genome. This framework, Systematic Training and Evaluation of Variant Evidence (STEVE), allows for the development of lab-specific models applicable to specific tests while permitting customization of the false positive capture rate settings to suit the requirements of the test.

## MATERIALS AND METHODS

### Overview

We performed cGS on the following GIAB Consortium samples with published truth sets: HG001–HG005.^11–13^ These sequence data were processed using two different secondary pipelines: Illumina’s Dragen Germline Pipeline^16^ and a pipeline consisting of alignment with Sentieon^17^ and variant calling with Strelka2.^18^ Each pipeline performed both alignment and variant calling to produce a Variant Call Format (VCF) file. Each VCF file was compared with the corresponding truth set to classify each variant call as a true positive call or a false positive call. Quality metrics for each variant call were extracted directly from the VCF file and converted into machine learning features. The variant calls were divided into six distinct data sets based on the variant type and genotype of the call. The six data sets were (1) SNV heterozygotes, (2) SNV homozygotes, (3) SNV complex heterozygous (two different nonreference alleles), (4) indel heterozygotes, (5) indel homozygotes, and (6) indel complex heterozygous (two different nonreference alleles). Each data set was used separately for training and testing of a machine learning model for that particular data type leading to six distinct models per pipeline, for a total of 12 models with the two pipelines we evaluated.

For each data set, we generally followed standard machine learning practices to create our models. For a primer on machine learning terminology in a medical context, we recommend Liu et al.^14^ We tested multiple freely available algorithms to train our models. The process included splitting the data set into training and testing sets, cross-validation, hyperparameter tuning, and a final evaluation on the testing set.^14,15^ We developed a set of clinical criteria required to pass a model, and developed a tie-breaking scheme when multiple models for a single data set were acceptable. Subsequently, we performed a retrospective analysis on a collection of variants identified through cGS that were previously orthogonally confirmed. Finally, we report on the clinical application of these models for nonactionable variants.

### Data set generation

All training and testing data sets for the machine learning models were derived from five, well-studied GIAB samples.^11–13^ Briefly, these samples consist of NA12878 (HG001), a well-studied female of European ancestry; HG002-004, a trio (son and parents) of Ashkenazi Jewish ancestry; and HG005-007, a trio (son and parents) of Chinese ancestry. HG006 and HG007 were only used for final testing. GIAB provides benchmark call regions, variants within those regions, and genotype calls for each variant for each of these five samples. Each benchmark region set covers 80–90% of reference genome hg38 and contains approximately 3.2–3.5 million, nonreference variant genotypes for the corresponding sample.^13^ We used the benchmark regions and variant calls from GIAB release v3.3.2.

DNA was purchased from Coriell or NIST (see Supplemental Materials) and sequenced with the Illumina NovaSeq 6000 sequencing platform. The DNA was sonicated, ligated to Illumina flowcell-specific unique dual-index adapters, and amplified using six cycles of polymerase chain reaction (PCR) and i5/i7 primers. The prepared library was then quality checked for adequate yield through fluorescence methods and quantitative PCR, as well as for appropriate library size and profile using bioanalysis. Libraries were clustered onto Illumina NovaSeq 6000 flowcells and sequenced using standard Illumina reagents and protocols. The output of this protocol was paired-end 150-bp reads in FASTQ format with a mean coverage of at least 30× and passing stringent quality control metrics.

The data were aligned to the human reference genome (hg38) and variants were called using Illumina’s Dragen Germline Pipeline (version 07.011.352.3.2.8b).^16^ Alignment and variant calling was also performed using a separate pipeline consisting of alignment with Sentieon (version 201808.07) and variant calling with Strelka2 (version 2.9.10).^17,18^ The output of each pipeline consisted of a single VCF file. These VCF files were matched with the corresponding GIAB benchmark regions and call sets and evaluated using the Real Time Genomic (RTG) vcfeval tool.^19^ RTG’s vcfeval can accept differences in variant representation and genotype differences while restricting the evaluation to only the benchmark regions. The final output consists of two VCF files per sample–pipeline combination, one containing the variants labeled as true positive calls and one containing the variants labeled as false positive calls.

We then converted the VCF files into machine learning labels and features. First, labels were assigned based on the RTG vcfeval output file. All variants in the true positive file were labeled as true positives, and all variants in the false positive file were labeled as false positives. Features were extracted directly from the VCF files as well. Generally, these were numerical values corresponding to quality metrics generated by the upstream pipeline. Importantly, the set of quality metrics available from each pipeline were different and shared metrics may be calculated differently due to implementation differences. Thus, the data from each pipeline were handled independently to create pipeline-specific models. We detail the precise set of features extracted for each pipeline in the Supplemental Material.

For each pipeline data set, we stratified all of the labels and features into one of six machine learning data sets based on the variant type and genotype combination. We used two categories for variant type (SNV or indel) and three categories for genotype (heterozygous, homozygous, and complex heterozygous [two different nonreference alleles]). Each of the six data sets was handled independently using an identical process that is detailed in the following section.

### Model training and testing

Our primary goal was the accurate identification of false positive variant calls. We also sought to minimize the number of true positive calls that would be labeled *incorrectly* as false positive calls. Since false positive variant calls (variants called by the pipeline but absent from the truth set) are the primary target, they are labeled as positives (binary label “1”) when passed to the machine learning algorithms. Similarly, true positive variant calls passed to the machine learning algorithm are labeled as negatives (binary label “0”). The goal of machine learning in this application is to create a model with high “capture rate” (sensitivity in our machine learning context), meaning that few or no false positive variant calls will be missed by the model and allowed onto the final patient report. The model should also have a low true positive flagging rate (“TP flag rate,” false positive rate in our machine learning context), meaning that the fewest true positive variant calls will be flagged by the model to be sent for confirmatory testing.

In general, we followed the machine learning guidelines recommended by Scikit-Learn (sklearn).^20^ The variants from each sample were divided into equal sized training and testing data sets such that the number of false positive and true positive variant calls were balanced. The testing data set was set aside for use in the final evaluation.

We selected four algorithms for model generation that each conformed to the sklearn paradigm: AdaBoost, EasyEnsemble, GradientBoosting, and RandomForest.^21–24^ We also selected hyperparameters for each model that were automatically evaluated during cross-validation (see below). See the Supplemental Material for further details concerning the hyperparameters evaluated.

We performed a leave-one-sample-out cross-validation using the training data.^14^ Given *S* samples, the models are trained on (*S*-1) samples then evaluated using the left-out sample to simulate receiving a “new” sample. This was performed a total of *S* times (each sample is left out once), leading to a sevenfold, leave-one-sample-out cross-validation in our analysis. As noted earlier, hyperparameters were automatically tested during the cross-validation process and the best performing hyperparameters (based on area under the receiver–operator curve) were used during the final training process.

Additionally, each model was evaluated at eight different capture rates in the range of 99–100%. These “evaluation” capture rates represent different thresholds that a clinical laboratory might select as a requirement for their test. Ninety-nine percent represents a capture rate that is likely at the lower end of acceptable practice (i.e., 1/100 false positive calls are missed) whereas 100% capture rate (i.e., *no* false positive calls missed) represents a clinical goal that is desirable but rarely achievable in practice. With six variant/call combinations, seven leave-one-sample-out cross-validation evaluations, and 45 model/hyperparameter combinations, a total of 1,890 models were trained during this process.

In the final step of the process, the models were retrained using only the best hyperparameters for each model and the full training set. Once trained, the models were then evaluated on the testing data set that was previously set aside. As noted earlier, each of these models was evaluated at eight different evaluation capture rates, leading to a total of 32 candidate hypertuned models for each variant/genotype data set.

### Clinical application

After the algorithms were trained, we developed a set of criteria to identify an algorithm to introduce into clinical practice. Given the results from the sevenfold cross-validation, we calculated both mean and standard deviation of each model’s capture rate. We defined the lower bound of capture rate as two standard deviations below the mean (-2 SD).

We then selected both a minimum acceptable capture rate and a target capture rate (i.e., the desired capture rate). Given those two values, two criteria were required for a model to pass: (1) the lower bound of the cross-validation capture rate (-2 SD) must be greater than or equal to the minimum acceptable capture rate and (2) the final testing capture rate must be greater than or equal to the lower bound of the cross-validation capture rate (-2 SD). The first requirement provides confidence that the trained models are consistently performing above the minimum acceptable capture rate. The second requirement provides confidence that the final trained model is consistent with the results from cross-validation and helps reject final models that are suffering from overfitting or underfitting. Because we had multiple evaluation capture rates, several models passed these two criteria. To break ties, we developed a modified F1 score that incorporates both the capture rate and specificity of the models to choose a single model for clinical use. Details of this implementation along with results from the trained models can be found in the Supplemental Material.

Given a set of accepted clinical models (one per variant/genotype combination), we used the models to perform a retrospective analysis of orthogonally confirmed variants that had been previously reported by the HudsonAlpha Clinical Services Lab (CSL). All variants were chosen from cGS cases reported by the CSL between 2 October 2019 and 11 December 2019. The variants chosen contained a mixture of primary findings, actionable secondary findings, carrier status findings, and pharmacogenomic findings. Each variant is associated with a VCF file that was generated using an identical process as the VCFs used in the model training. Finally, we report the results of this approach in clinical practice, applied to carrier status findings and pharmacogenomic findings. Variants that were primary findings or actionable secondary findings were sent for confirmatory testing regardless of the model’s predictions. Carrier status findings and pharmacogenomic findings were sent for confirmatory testing when the model predicted the variant to be a false positive, indicating that the trained model did not have high confidence in the variant call.

## RESULTS

### Variant collection

HG001 (NA12878) was sequenced with three replicates and HG002 through HG005 were each sequenced once. The number of variants called across all samples was greater than 24 million true positive calls with 137 thousand false positive calls using the Dragen pipeline. Details of these counts by sample, variant type, and genotype along with a detailed description of the pipeline and RTG vcfeval invocations is available in the Supplemental Material.

### Model evaluation

For our model selection and evaluation, we chose a minimum acceptable capture rate of 99% (indicating 1/100 false calls are missed) with a target capture rate of 99.5% (indicating 1/200 false calls are missed). Given these criteria, a number of models passed the evaluation process. The best model was chosen using the modified F1 score described above. The results for the final chosen models for all six variant–genotype combinations are shown in Table 1. Additional information for all final trained models at each evaluation capture rate is available in the Supplemental Material.

**Table 1:** Summary of trained models for Dragen-based pipeline.

Variant/genotype	Best model	CV capture rate (%)	Final capture rate (%)	CV TP flag rate (%)	Final TP flag rate (%)
SNV—heterozygous	GradientBoosting	99.76 + −0.18	99.58	12.78 + −2.26	12.20
SNV—homozygous	EasyEnsemble	99.94 + −0.14	99.75	17.25 + −2.07	17.40
SNV—complex heterozygous	—	—	—	—	—
Indel—heterozygous	GradientBoosting	99.62 + −0.26	99.68	43.11 + −3.35	43.41
Indel—homozygous	GradientBoosting	99.78 + −0.27	99.50	55.65 + −4.16	55.16
Indel—complex heterozygous	GradientBoosting	99.86 + -0.14	99.60	53.45 + −5.65	54.22

For each variant–genotype combination, the following table reflects the best model for our criteria, the cross-validation (CV) mean and standard deviation for capture rate and true positive (TP) flag rate, and final evaluation for capture rate and TP flag rate.

*SNV* single-nucleotide variant.

Five of the six variant–genotype combinations had at least one model passing our criteria. The only failing combination was complex heterozygous SNVs (two nonreference alleles in *trans* at the same position), a failure that is likely due to the rarity of such events (45 false positive calls across all seven samples). Note that in our approach, any failing combination indicates a variant/genotype class that will always be sent for confirmation testing. Models that were selected for use in clinical practice had a final capture rate that was greater than or equal to our chosen target capture rate of 99.5%.

We also tested the final models against two data sets (HG006 and HG007) that were not used during training, cross-validation, or the final testing process. We limited the scope of this experiment to exonic regions overlapping the benchmark regions for the sample. Overall, the false positive (FP) capture rate was 99.70% (331/332) with a TP flag rate of only 12.99% (26,924/207,339). Details of the HG006 and HG007 analysis, technical challenges involving the GIAB benchmark regions, and the performance by variant type are described in the Supplemental Materials.

### Clinical evaluation

As we developed the models, we tracked Sanger confirmation results for cGS cases. The indication for testing was rare, undiagnosed diseases. The first phase of the clinical evaluation was a retrospective analysis of recent cases for which Sanger confirmation results were available. We collected the orthogonal testing results for 232 variants from 26 cGS cases and compared them with the predictions from the Dragen-trained models. Note that this data set includes all variants that were sent for confirmation including primary or actionable variants. The results of this retrospective analysis are seen in Table 2. Only two variants in this data set failed to confirm by orthogonal testing. Both were predicted to be false calls by the models. Of the 230 remaining true positive calls (i.e., confirmed by orthogonal testing), only 36 were incorrectly flagged as false positives by the models. This indicates an observed TP flag rate of 15.58% with observed model-specific TP flag rates ranging from 2.94% to 25.00%.

**Table 2:** Summary of retrospective variant analysis.

Variant/genotype	Confirmed true calls	False calls	False calls captured	True calls flagged	Model TP flag rate
SNV—heterozygous	176	0	—	29 (16.48%)	12.20%
SNV—homozygous	34	0	—	1 (2.94%)	17.40%
SNV—complex heterozygous	0	0	—	—	—
Indel—heterozygous	20	2	2 (100.00%)	5 (25.00%)	43.41%
Indel—homozygous	0	0	—	—	55.16%
Indel—complex heterozygous	0	0	—	—	54.22%

Here we report the total number of variants confirmed to be true positive (TP) or false positive calls, the number of false positive calls correctly identified (capture rate), and the number of true calls incorrectly labeled as false calls (TP flag rate). The model TP flag rate (i.e., expected TP flag rate) from the final evaluation is also provided here for comparison. Models used for this analysis were generated from the Dragen-based pipeline.

*SNV* single-nucleotide variant.

Following the development and evaluation noted above, we employed the models in clinical practice to reduce the number of Sanger confirmations that were ordered in subsequent genome sequencing cases. As noted earlier, these models were only applied to nonactionable variants (carrier status findings and pharmacogenomic findings). Primary and actionable secondary variants continued to be sent for Sanger confirmation and were therefore excluded from this analysis. Additionally, every qualifying variant call that was predicted to be a true positive was manually reviewed using the Integrative Genomics Viewer^25^ as an additional review of the model’s prediction. We have applied the prediction algorithm to 252 nonactionable variants from 31 cGS cases gathered from the Dragen-based pipeline. Application of these models reduced the number of variants that had orthogonal confirmation by 216 (85.71%) overall, with an average reduction of 7 variants per sample. Sanger confirmation testing generally costs at least $100 (USD) per variant indicating an average cost savings of $700 per sample. Analysis of these results by variant type and genotype is shown in Table 3.

**Table 3:** Summary of prospective variant predictions.

Variant/genotype	Predicted false positive calls	Predicted true positive calls	Orthogonal order reduction
SNV—heterozygous	29	164	84.97%
SNV—homozygous	1	34	97.14%
SNV—complex heterozygous	0	0	—
Indel—heterozygous	6	18	75.00%
Indel—homozygous	0	0	—
Indel—complex heterozygous	0	0	—
Overall	36	216	85.71%

This table details the outcome of the use of the models in clinical cases. It shows the total number of variants that were predicted to be false positive or true positive in the clinical cases along with the percentage of variants that were not sent for orthogonal confirmation.

*SNV* single-nucleotide variant.

We analyzed these prospective cases by applying the model to four variant subsets along two risk axes: (1) risk of a false positive having an adverse effect on patient care and (2) risk of reporting a false positive variant call. First, if the models are applied to actionable variants, there is increased risk of a false positive affecting patient care because there is a candidate treatment or therapy that may be erroneously applied. Second, if the models are applied to variants outside the GIAB benchmark regions, there is increased risk of reporting a false positive variant call because the models may not be trained to handle complexities from excluded regions such as structural variants, tandem repeats, etc.^11–13^ Thus, there is reason to believe that variant calls outside of *all* of the GIAB benchmark regions will be systematically different from those inside the benchmark regions. As with Lincoln et al.,^10^ we selected the union of the benchmark regions to prevent an individual GIAB sample from excluding a benchmark region.

Given the above risk axes, we define the four subsets of all reported variants (*n* = 306) from each case: (1) all variants, (2) variants within the union of GIAB benchmark regions, (3) nonactionable variants, and (4) variants that are both nonactionable and within the union of GIAB benchmark regions. Given these risk categories, applying the models to all variants (approach 1) results in the lowest rate of confirmatory testing (21%), but also has the highest risk of reporting false positive variants that may adversely impact patient care. The most conservative approach (approach 4) results in the highest rate of confirmatory testing (54%), but also has the lowest risk of reporting false positive variants that may adversely impact patient care. Our chosen approach (approach 3) maintains a low rate of confirmatory testing (29%) while still requiring confirmatory testing for all actionable variants. The full data for each of these approaches are shown in Table 4.

**Table 4:** Summary of clinical approaches.

Category	All variants	GIAB benchmark only	Nonactionable only	GIAB benchmark + nonactionable only
Risk of reporting false positive	Higher	Lower	**Higher**	Lower
Risk of adverse impact on patient care	Higher	Higher	**Lower**	Lower
Not eligible—no passing model	6	6	**6**	6
Not eligible—outside benchmark region AND actionable	N/A	N/A	**N/A**	4
Not eligible—actionable	N/A	N/A	**48**	44
Not eligible—outside benchmark region	N/A	88	**N/A**	84
Eligible—predicted true	240	164	**216**	141
Eligible—predicted false	60	48	**36**	27
Confirmation order rate	21.57%	46.41%	**29.41%**	53.92%

This table summarizes the prospective results for all variants (*n* = 306) under different clinical approaches. The methods are organized from highest risk of reporting a false positive to lowest, where reporting actionable variants without confirmation is considered highest risk. Approaches that allow the models to be applied to any variant interpretation (specifically primary or actionable) have a higher risk for adverse impact on patient care. Approaches that allow for variants from any genomic region (specifically outside Genome in a Bottle [GIAB] benchmark regions) have a higher risk of reporting a false positive. Variants that are classified as “not eligible” either did not have a validated model or require confirmation test due to the approach. Confirmation order rate is the percentage of variants that are either not eligible or predicted false, indicating that a confirmation test would be ordered for that variant prior to reporting. The results from our clinical approach (nonactionable only) are emphasized.

### Pipeline comparison

In addition to the Dragen-based pipeline, we ran a second pipeline consisting of Sentieon and Strelka2. Over 24 million true positive calls with 419 thousand false positive calls were found using the Sentieon/Strelka2 pipeline (a threefold increase in false positive calls relative to Dragen-based pipeline). The details of this pipeline, the RTG vcfeval results, and training/testing results are all available in the Supplemental Materials.

The models trained on Sentieon/Strelka2 data showed a marked improvement over those trained for the Dragen pipeline. While maintaining the 99.5% target capture rate, there was a decrease in TP flag rate across all model types except complex heterozygous indels. The important differences include a 1.62% TP flag rate for heterozygous SNVs compared with 12.20% for the Dragen pipeline and 20.29% TP flag rate for heterozygous indels compared with 43.41% for the Dragen pipeline. These differences are explained, in part, by the increase in total false positive calls relative to Dragen. This suggests that despite the overall decreased precision of the Sentieon/Strelka2 pipeline, the models are able to identify false positives more easily for the Sentieon/Strelka2 pipeline compared with the Dragen pipeline.

Given the relative ease of identifying false positives, we tested a version of the models with very stringent criteria: a minimum capture rate of 99.9% and a target capture rate of 100%. These stringent models with the Sentieon/Strelka2 pipeline resulted in a final TP flag rate of 28.02% for heterozygous SNVs. In comparison, the stringent models with the Dragen pipeline had a very high final TP flag rate of 88.05% for heterozygous SNVs, a rate that severely limits its usefulness. Results for all stringent models for both pipelines are in the Supplemental Material.

## DISCUSSION

We developed a framework for training models to identify false positive variant calls from genome sequencing data sets. Our approach advances that of Lincoln et al.^10^ by using numerical values (rather than flags) as feature inputs to machine learning models. Additionally, by using clinical genome sequencing data instead of targeted sequencing, we increased the total number of true positive and false positive calls that were available for training and testing. This data obviates the need for a large set of orthogonal test results, a resource that is not available to most laboratories. In addition, the final models are tunable, allowing laboratories to adjust the minimum and target capture rates of the models to values that are relevant to a particular intended use of a test. Furthermore, the framework we developed can be used in conjunction with a variety of upstream pipelines as shown by the two different aligner–caller combinations used in this study. Custom models can also be developed to match upstream processes different from those used in this study such as different sequencing technologies, different secondary pipelines, and different versions of the software used in a pipeline.

The differences in model results between the two tested pipelines suggest that the upstream pipeline significantly influences the final performance of the models. For example, the final TP flag rates (the fraction of true variants that would be flagged for orthogonal confirmation) were all lower with the Sentieon/Strelka2 pipeline compared with the Dragen pipeline with one exception. This suggests that while Sentieon/Strelka2 is generating more false positive calls (i.e., reduced precision compared with the Dragen pipeline), the features extracted from the VCF were better able to differentiate false positive calls from true positive calls compared with the features produced by the Dragen pipeline (see Supplemental Material). We anticipate that other informatic pipelines will have similar variability in performance. Therefore we recommend building custom models for each different upstream pipeline used. Note that these differences may include any modification of the steps from the sample extraction to the final variant file, for example: sample type (blood/saliva), extraction technique, library preparation, sequencing method, de-multiplexing method, read trimming, read correction, alignment (mapping), marking or removing duplicate reads, base quality recalibration, variant calling, batch calling, variant annotation steps, and variant filtering steps.

We note that these differences between pipelines are not unique to our approach. While our methodologies were different from Lincoln et al.,^10^ they are similar enough to make some observations regarding the different pipelines. For SNVs from Lincoln et al., lab 1 had a confidence interval lower bound on the capture rate of 98.9% while only flagging 4.1% of true positive variants. We compare this with the -2 SD from our cross-validation capture rate (noting that these values are not exactly comparable), which was 99.4% for heterozygous SNVs and 99.66% for homozygous SNVs while flagging 13.16% of our true positive clinical SNVs. The difference in capture rates may be attributed to the limited number of false positives available to Lincoln et al. (fp = 211) compared with our data (fp = 34,890). This suggests that some applications of these approaches will be limited by the test itself due to a low occurrence of false positives that can be used for training.

It appears that our algorithm does not perform as well as that of lab 1 in the Lincoln et al. study^10^ with respect to indels. Lab 1 has 992 false positives. This shifts the lower bound on capture rate up to 99.8% with a TP flag rate of 6.7%. This can be compared with our -2 SD of 99.1% for heterozygous indels (fp = 215,261) and 99.24% for homozygous indels (fp = 31,410). Our algorithm flags a greater number of our true positive clinical indels, 25%. While we cannot rule out methodology as a contributing factor, the bulk of this difference may be attributed to differences in the upstream pipelines from both a data and processing perspective. First, our data itself is likely far more diverse than that of either lab in the Lincoln et al. study. Since we used cGS, we were only constrained to the benchmark regions provided by GIAB for variant selection leaving us with a 200-fold increase in false positive indels for training and testing. This means our data set includes many genomic contexts that are likely absent or underrepresented in the Lincoln et al. data set such as noncoding exons, introns, intergenic regions, pseudogenes, and short-tandem repeats. Second, we did not perform any post–variant calling filtering (manual or automatic) of our variants prior to training the models. As a result, any variant call reported by the calling software that was in a benchmark region was used in our analysis. In contrast, Lincoln et al.’s lab 1 performs an automated and manual filtering process to remove some “clearly false variant calls.”^10^ Lincoln et al. suggest that this likely makes a major difference compared with their lab 2 results. Lab 2 had less stringent automated filtering and a limited or absent manual review process on their data. However, their results were also comparably worse (lower bound of capture rate = 99.1%, TP flag rate = 15.4% for indels). Limiting the scope of our genome sequencing data to variants that pass a filtering criteria (preferably automated due to the scale of our data) or that reside only in specific genomic contexts (e.g., gene regions) may reduce the complexity that the models need to capture and ultimately improve the performance. However, these specialized or focused models are less likely to be generally applicable to any variant from genome sequencing, and they would require more samples to reach the same number of data points for training/testing due to the filtering component of the upstream pipeline.

Additional annotations to called variants may also improve the performance of the trained models. For example, a feature indicating strand bias was of great importance in the Sentieon/Strelka2 heterozygous SNV model, but that feature is not available in the Dragen pipeline outputs. Adding this feature to the Dragen VCF could improve the model performance. Furthermore, almost all of the features we used are not tied to the variant itself but are tied to the call (i.e., genotype, call quality, strand bias, etc.). Adding positional or variant annotations may be beneficial by providing the algorithms with features that are associated with genomic context such as reference/alternate allele, measures of local genomic complexity (i.e., low-complexity or short-tandem repeat), or pseudogene presence. This additional information may make the individual variant calls more identifiable and could lead to an overfitting scenario where the model ignores quality information and has instead learned to recognize a particular variant, regardless of its quality. Of course, models that have learned to recognize problematic areas of the reference genome may prove to be desirable in practice. Finally, studying the false positives calls that are currently missed by the trained models may provide evidence supporting the inclusion of some of the features discussed above.

Our results also suggest that there are differences between the full set of variants used in training and the set of variants that we are currently sending for orthogonal confirmation. For example, both heterozygous SNVs and indels had observed TP flag rates that are relatively close to the expected TP flag rate of the model (see Table 2). In contrast, homozygous SNVs had an observed TP flag rate (2.94%) much lower than the expected TP flag rate from model evaluation (17.40%). While the numbers are too small to meet statistical significance, the results suggest that reported homozygous SNV variants are more likely to be predicted as true positives than homozygous SNV variants chosen at random. More data will be needed to assess this trend and determine whether similar trends occur for other variant types.

There are limitations and potential drawbacks to our approach. First, our approach is not trained on orthogonal results generated by Sanger sequencing. Instead, we trained on the benchmark regions for five samples provided by GIAB, which offers a diverse set of variation across different genomic contexts and backgrounds. Note that this is far more variation than can be feasibly acquired through orthogonal, confirmatory testing for an individual laboratory and includes more variants than those that are clinically relevant. However, despite this major improvement in available training variants from GIAB, the training data set is still limited in scope to the GIAB benchmark regions. As discussed earlier, variant calls outside the GIAB benchmark regions may be systematically different from those inside the benchmark regions. We recommend that clinical laboratories initially apply algorithms such as those described in this paper to the lowest risk variants (only nonactionable variant calls inside GIAB benchmark regions) and only consider other approaches after careful evaluation and additional studies.

There may also be other sources of bias as a result of using the GIAB data sets for training. A complete understanding of the weight of each feature in a machine learning model is very difficult, but is important when the result may impact patient safety.^15^ While there are tools in place to aid in the interpretation of some models, they do not apply to all of the models we trained. The Supplement Materials detail some interpretation information referred to as “feature importances,” denoting which features are most influential in the models.^20^

As recommended in Rehm et al.,^3^ this study demonstrates the need to understand the intricacies in technologies and bioinformatics before eliminating orthogonal confirmation. In addition to being cognizant of the pipeline differences and limitations listed above, a laboratory must make several choices to apply the algorithm developed here, notably the inherent trade-off between risk of reporting a false positive and cost of confirmatory testing. We presented the results of four approaches applied in our prospective analysis. Each has a different level of risk and associated confirmatory testing cost. Because this method is intended to be used with patient samples, the study was performed in concordance with professional and lab accreditation standards for clinical testing.^3,26^

We chose to apply the algorithm only to nonactionable findings, with a clear description in the report of which variants required confirmation as recommended by the ACMG standards.^3^ This approach has a relatively low risk of an adverse patient outcome. However, our approach does have a higher risk of identifying a false positive variant that will be proven a false positive by orthogonal testing because we are applying it to nonactionable variants outside the GIAB benchmark regions. We selected this approach because it substantially reduced the overall confirmatory testing rate to 29% without compromising patient safety (see Supplemental Materials for a discussion of variants outside GIAB benchmarks). Notably, if we had selected the most conservative approach, we still would have observed a 47% reduction in confirmatory testing.

Several variables are relevant to the actual training and testing of the models. The minimum and target capture rates of the models are the most important. Increasing these values will capture more false positive variant calls, but will also increase the number of true calls that are flagged as false positives. This is appropriate for applications such as actionable variants where stricter capture rates are required. Additionally, setting different minimum and target capture rates based on the variant type may be appropriate. For example, indels were consistently the major type of false positive call despite having far fewer total calls than SNVs. Within genes, indels are more frequently interpreted as pathogenic due to variant types such as frameshifts. A clinical laboratory may therefore choose to impose stringent requirements on the capture rates for indel models to reflect the pathogenicity of the variant and/or the increased relative likelihood that any given variant is a false positive.

Whether to restrict the data used for model training should also be considered. We used the complete data from GIAB benchmark regions for model training, providing the maximum amount of benchmark variant calls to the models. Nevertheless, other laboratories may wish to limit the analysis to particular regions (e.g., gene or coding regions) or to exclude difficult regions (e.g., short-tandem repeats, homopolymer regions, or pseudogenes) from the data set. This reduces the number of variants the models can use for training and testing, but it also reduces the complexity that the models are capturing. This may result in more specialized models that perform better for those subsets of variants. Additionally, these specialized models may be better suited for stringent target capture rates due to the reduction in complexity of the input variants.

The choice of samples and cross-validation method can also impact the final results. In our primary training, we tested the models with three replicates of HG001 and one replicate of HG002-HG005 using a leave-one-sample-out approach for cross-validation. While we do not suspect bias in the data due to the unidentified variant features used by the models, it cannot be ruled out without further experimentation. Adding additional replicates of HG002-HG005 would likely improve the results by reducing any subtle biases and adding more data points to training and testing. Alternate methods for cross-validation should also be considered such as classic training–testing splits.^14^

While they were different in development, our results and those found in Lincoln et al.^10^ have many similarities. First, many of the features (or “flags” in Lincoln et al.) are conceptually similar, even if calculated differently. In particular, allele depth, allele frequency, call quality, and presence of nearby variants were fundamental in both approaches. Second, we both observe that the choice of upstream pipeline has a major impact on the final model’s performance. We demonstrated major differences when comparing the Dragen pipeline to Sentieon/Strelka2. Similarly, Lincoln et al. demonstrated how filtering can influence the results derived from lab 1 and lab 2. Third, we both train separate models for different data types. We separated by variant type (SNV or indel) and genotype, whereas Lincoln et al. only separated by variant type. In both approaches, the performance of the model for each data type varied and the relative feature importances also changed. In some instances, a feature was very important in one of our models, but not used in another model. Finally, across all pipelines in both our data and Lincoln et al., false positive indels were consistently more challenging to identify than SNVs. This is most obvious when reviewing the TP flag rates, which indicates a higher fraction of true indel calls are being flagged for confirmation than true SNV calls across all approaches. This suggests that indels are more difficult to capture across our current pipelines, or that a key feature for distinguishing false positive indels is missing from our annotations. Despite differences in the underlying data and approaches, these similarities suggest that the general approach of using GIAB data along with variant quality metrics to train models is one that is broadly applicable to many different clinical genomic tests. These studies show that the cost and time burdens of orthogonal confirmation testing can be focused on a carefully chosen subset of variants without adverse impacts on test quality or patient safety.

## Supplementary information

Supplemental Document

## Data Availability

All code used to generate our models and results is available in STEVE v0.1.0, which is publicly available at https://github.com/HudsonAlpha/STEVE. It is available for use under the Apache 2.0 License.
