# Supplementary material for: Reducing Sanger confirmation testing through false positive prediction algorithms
Source: Genet Med. 2021 Mar 25;23(7):1255–62. doi: 10.1038/s41436-021-01148-3 (PMC8257489; doi:10.1038/s41436-021-01148-3)
Supplement: Supplementary file 1 — Supplemental Document [file 41436_2021_1148_MOESM1_ESM.pdf]

Supplemental Document

J. Matthew Holt et al.

December 14, 2020

Contents

1 Sample metadata **3**
1.1 General Sample Info . . . 3
1.2 Samples . . . 3

2 Genome Sequencing Pipelines **4**
2.1 Dragen Pipeline . . . 4
2.1.1 Integrated Command . . . 4
2.2 Sentieon / Strelka2 Pipeline . . . 4
2.2.1 Sentieon paired-end alignment . . . 4
2.2.2 Sentieon deduplication . . . 5
2.2.3 Sentieon Base Quality Recalibration . . . 5
2.2.4 Strelka2 Variant Calling . . . 6
2.3 RTG VCFeval Analysis . . . 7

3 Model-Training Pipeline **8**
3.1 Feature Extraction . . . 8
3.2 Model Hyperparameters . . . 9
3.3 Clinical Model Selection Formula . . . 9

4 Results for dragen-07.011.352.3.2.8b/dragen-07.011.352.3.2.8b **11**
4.1 RTG vcfeval Results . . . 11
4.1.1 Pipeline Performance . . . 11
4.1.2 Variant Counts . . . 11
4.2 Model Results . . . 11
4.2.1 Selected Models . . . 11
4.2.2 Strict Models . . . 11
4.2.3 Feature Importances . . . 14
4.2.4 Model for SNV-HET . . . 14
4.2.5 Model for SNV-HOM . . . 15
4.2.6 Model for SNV-HE2 . . . 16
4.2.7 Model for INDEL-HET . . . 16
4.2.8 Model for INDEL-HOM . . . 17
4.2.9 Model for INDEL-HE2 . . . 18

5 Results for sentieon-201808.07/strelka-2.9.10 **19**
5.1 RTG vcfeval Results . . . 19
5.1.1 Pipeline Performance . . . 19
5.1.2 Variant Counts . . . 19
5.2 Model Results . . . 19
5.2.1 Selected Models . . . 19
5.2.2 Strict Models . . . 19

5.2.3 Feature Importances . . . 21
5.2.4 Model for SNV-HET . . . 21
5.2.5 Model for SNV-HOM . . . 23
5.2.6 Model for SNV-HE2 . . . 23
5.2.7 Model for INDEL-HET . . . 24
5.2.8 Model for INDEL-HOM . . . 25
5.2.9 Model for INDEL-HE2 . . . 25

6 HG006 and HG007 Experiment **27**

6.1 Datasets . . . 27
6.2 RTG vcfeval Performance . . . 27
6.3 Observations from the HG006 and HG007 experiments . . . 27
6.3.1 Background . . . 27
6.3.2 Different Processing Date . . . 28
6.3.3 Benchmark Regions . . . 28
6.3.4 Clustered False Positives . . . 29
6.3.5 Manual inspection of one uncaptured run region . . . 30
6.3.6 A possible explanation of our findings . . . 31
6.3.7 Addressing the issues with HG006 and HG007 . . . 31
6.4 Additional False Positive Information . . . 31
6.5 Final Performance on HG006 and HG007 . . . 34

7 Notes on application outside benchmark regions **34**

1 Sample metadata

1.1 General Sample Info

This section contains information regarding where samples were acquired from. This corresponds to the "Sample" label in Table 1.

1. NA12878 - female of European ancestry; purchased through `https://www.coriell.org/0/Sections/Search/Sample_Detail.aspx?Ref=NA12878&Product=DNA`
2. HG002-HG004 - son and parents of Eastern Europe Ashkenazi Jewish ancestry; purchased through `https://www-s.nist.gov/srmors/view_detail.cfm?srm=8392`
3. HG005 - male of Chinese ancestry; purchased through `https://www-s.nist.gov/srmors/view_detail.cfm?srm=8393`

1.2 Samples

This section contains information regarding the specific samples used for analysis. This data is automatically pulled from a sample JSON file containing sample names, sample types (i.e. which GIAB sample), and how the sample was prepared. Table 1 contains the list of metadata as pulled from the JSON.

Note: HG006 and HG007 were used only for testing.

Library	Sample	Preparation	Mean Coverage
SL362490	NA12878	Clinical PCR	34.57
SL362491	NA12878	Clinical PCR	34.44
SL362492	NA12878	Clinical PCR	37.83
SL409548	HG002	Clinical PCR	33.66
SL409549	HG003	Clinical PCR	36.57
SL409550	HG004	Clinical PCR	30.12
SL409551	HG005	Clinical PCR	32.01

Table 1: This table contains metadata regarding each sequenced sample. The GIAB sample label and prep type are currently the two pieces of tracked metadata regarding each sample.

2 Genome Sequencing Pipelines

2.1 Dragen Pipeline

Illumina's DRAGEN platform is a rapid genome analysis platform that performs both alignment and variant calling steps using hardware acceleration. The details of this platform can be found on Illumina's DRAGEN webpage.

2.1.1 Integrated Command

Because the Dragen solution is fully integrated from FASTQ to gVCF, there is only one command we used to collect the final gVCFs. The final result of this step is the hard-filtered gVCF file (and corresponding index file) of the format `${sample}.hard-filtered.gvcf.gz`. That gVCF file is given to RTG VCFeval for variant evaluation.

```
dragen -f \
    -r /staging/reference/hg38/hg38.fa.k_21.f_16.m_149 \
    --fastq-list /staging/fastq/${sample}_fastqs/${sample}_list.csv \
    --bin_memory 60000000000 \
    --output-directory /staging/bam/ \
    --output-file-prefix ${sample} \
    --enable-duplicate-marking true \
    --enable-map-align-output true \
    --enable-variant-caller true \
    --vc-sample-name ${sample} \
    --vc-emit-ref-confidence GVCF \
    --dbsnp /staging/reference/hg38/dbsnp_146.hg38.vcf
```

2.2 Sentieon / Strelka2 Pipeline

This pipeline uses a combination of Sentieon (more efficient implementation of BWA-mem) for alignment and Strelka2 for variant calling. The pipeline is implemented using a snakemake workflow, and relevent commands are presented in order below. All parameters referring to a reference genome are using the hg38 reference genome with ALT contigs.

2.2.1 Sentieon paired-end alignment

The following command is used on each pair of FASTQ files for a sample. In brief, it performs the alignment process using sentieon, passes that into the post-alt alignment process derived from bwa-kit (this is recommended due to ALT contigs in the hg38 reference), and finally used the sention sorting function. The output of this command is a single, position-sorted BAM file that has been post-alt processed and the corresponding index file.
Parameters:

1. rgoptions - Read Group (RG) options for the particular flowcell/lane combination
2. reference - the filename for the reference genome (hg38 with all ALT contigs for our use case)
3. bwakit - directory containing a download of the bwa-kit post-ALT processing
4. tempParams - a temporary directory, can be removed without altering command outputs

```
sentieon \
    bwa mem -M \
    -R "{params.rgoptions}" \
    -t {threads} \
    -K 10000000 \
```

```
    {params.reference} \
    {input.fq1} {input.fq2} | \
{params.bwakit}/k8 \
    {params.bwakit}/bwa-postalt.js \
    {params.reference}.alt | \
sentieon util sort {params.tempParams} \
    --bam_compression 1 \
    -r {params.reference} \
    -o {output.bam} \
    -t {threads} \
    --sam2bam \
    -i -
```

2.2.2 Sentieon deduplication

The following command will gather duplication statistics across *all* BAM files for a sample and then simultaneously remove duplicates while merging the BAM files together. The output of this step is a single BAM file containing all alignments for the sample and the corresponding index file.

Parameters:

1. sortedbams - this is a concatenation of `-i {BAM}` for each BAM file in the sample (i.e. each flowcell/lane BAM file generated in the previous step)

```
sentieon driver \
    -t {threads} \
    {params.sortedbams} \
    --algo LocusCollector \
    --fun score_info \
    {output.score} && \
sentieon driver {params.tempParams} \
    -t {threads} \
    {params.sortedbams} \
    --algo Dedup \
    --rmdup \
    --score_info {output.score} \
    --metrics {output.metrics} \
    --bam_compression 1 \
    {output.dedupbam}
```

2.2.3 Sentieon Base Quality Recalibration

The following command will gather base quality score information for the de-duplicated sample BAM file and then perform base quality score recalibration (BQSR) on the BAM. The output of this step is a single BAM file containing the recalibrated mappings for the sample and the corresponding index file. This is the final BAM file for the sample.

Parameters:

1. reference - the filename for the reference genome (hg38 with all ALT contigs for our use case)
2. dbsnp - this is the dbSNP file gathered from this URL: `ftp://gsapubftp-anonymous@ftp.broadinstitute.org/bundle/hg38/dbsnp_146.hg38.vcf.gz`
3. mills - the is the Mills indel file gathered from this URL: `ftp://gsapubftp-anonymous@ftp.broadinstitute.org/bundle/hg38/Mills_and_1000G_gold_standard.indels.hg38.vcf.gz`
4. tempParams - a temporary directory, can be removed without altering command outputs

```
sentieon driver \
    -r {params.reference} \
    -t {threads} \
    -i {input.dedupbam} \
    --algo QualCal \
    -k {params.dbsnp} \
    -k {params.mills} \
    {output.recaltable} && \
sentieon driver {params.tempParams} \
    -r {params.reference} \
    -t {threads} \
    -i {input.dedupbam} \
    -q {output.recaltable} \
    --algo ReadWriter \
    {output.recalbam}
```

2.2.4 Strelka2 Variant Calling

The following command will execute the Strelka2 workflow to perform variant calling. As an additional step, we annotate the final VCF file from Strelka2 with dbSNP identifiers (this is primarily for QC purposes in the pipeline). The final result of this step is a VCF file with dbSNP identifiers and the corresponding index file. This is the final VCF file that is provided as input to RTG VCFeval.
Parameters:

1. strelka - the path to the repo contain strelka2
2. reference - the filename for the reference genome (hg38 with all ALT contigs for our use case)
3. contigs - this is a restricted contig file (BED format) to reduce run time of Strelka2, see README file at `https://github.com/Illumina/strelka/blob/v2.9.x/docs/userGuide/README.md#improving-runtime-for-refe` for the exact file and context behind usage
4. memGB - a memory limit for strelka2
5. bcftools - path to a bcftools executable for performing annotation; the version used was 1.10.2
6. dbsnp - this is the dbSNP file gathered from this URL: `ftp://gsapubftp-anonymous@ftp.broadinstitute.org/bundle/hg38/dbsnp_146.hg38.vcf.gz`

```
{params.strelka}/bin/configureStrelkaGermlineWorkflow.py \
    --bam {input.bam} \
    --referenceFasta {params.reference} \
    --callRegions {params.contigs} \
    --runDir {output.runDir} && \
{output.runDir}/runWorkflow.py \
    -m local \
    -j {threads} \
    -g {params.memGB} && \
{params.bcftools} annotate \
    -a {params.dbsnp} \
    -c ID \
    -O z \
    -o {output.vcf} \
    {output.runDir}/results/variants/variants.vcf.gz && \
tabix {output.vcf}
```

2.3 RTG VCFeval Analysis

This tool was used to label variant calls as either true or false positives depending on presence or absence from the corresponding GIAB truth set. Note that these variants are limited to those found within the GIAB high-confidence regions (i.e. variants outside those regions are excluded).
Parameters:

1. truth - this is the VCF of variants published by GIAB representing a sample's truth set
2. bed - this is the high-confidence regions published by GIAB for the truth set; variants outside these regions are NOT evaluated
3. sdf - a file format required by RTG VCFeval (build from the hg38 reference)

```
rtg vcfeval \
    --all-records \
    -b {params.truth} \
    -c {input.vcf} \
    --bed-regions {params.bed} \
    -t {params.sdf} \
    -T {threads} \
    -o {output.rtgDir}
```

3 Model-Training Pipeline

This section contains details related to the methodology used for training all models. Note: an identical process is used for each pipeline, allowing for different configuration of inputs depending on the upstream pipeline.

3.1 Feature Extraction

While an identical process is used for each pipeline, the features from each pipeline are configurable using a combination of JSON and hard-coded Python3 (when complex features are involved). Features must be numerical values when given to the models, so some transformations are necessary from the raw VCF specified values.

In the file `{REPO}/scripts/model_metrics.json`, there are a list of features defined for different upstream callers. When a feature is practically copied from a VCF file, we try to denote it below with the corresponding VCF tag. Here is a brief description of the sub-types and features (Note: not all types are used in each pipeline):

1. "CALL" - These features are generally tied to a genotype call (i.e. sample-specific)
   (a) AD0 - the allele depth (AD) for the first allele in the genotype (e.g. if GT=0/1, this is the depth of the reference allele)
   (b) AD1 - the allele depth (AD) for the second allele in the genotype (e.g. if GT=0/1, this is the depth of the first alternate allele)
   (c) ADO - the total allele depth (AD) for any alleles that are not present in the genotype call
   (d) AF0 - the allele frequency for the first allele in the genotype (e.g. if GT=0/1 and AD=10,30 then this value is 0.25)
   (e) AF1 - the allele frequency for the second allele in the genotype (e.g. if GT=0/1 and AD=10,30 then this value is 0.75)
   (f) AFO - the total allele frequency for any alleles that are not present in the genotype call
   (g) GT - the genotype field (GT) transformed into a single numerical value
   (h) DP - the depth field (DP)
   (i) GQ - the genotype quality (GQ) field
   (j) DPI - the indel read depth (DPI)
   (k) GQX - empirically calibrated genotype quality score (GQX)
   (l) DPF - basecalls filtered prior to genotyping (DPF)
   (m) SB - sample site strand bias (SB)
2. "INFO" - These features are generally tied to a variant site and may represent aggregate quality statistics in multi-sample VCF files (i.e. variant-specific metrics)
   (a) DB - represents dbSNP membership (DB)
   (b) FractionInformativeReads - fraction of informative reads out of the total reads (FractionInformativeReads)
   (c) FS - Phred-scaled Fisher's Exact Test for strand bias (FS)
   (d) MQ - mapping quality (MQ)
   (e) MQRankSum - rank sum test for mapping qualities (MQRankSum)
   (f) QD - variant confidence by depth (QD)
   (g) R2_5P_bias - score based on mate bias and distance from 5-prime end (R2_5P_bias)
   (h) ReadPosRankSum - measure of position bias (ReadPosRankSum)

(i) SOR - measure of strand bias using contingency table (SOR)

(j) SNVHPOL - SNV context homopolymer length (SNVHPOL)

3. "MUNGED" - These features are generally calculated from information present in the VCF files that does not cleanly fall into either the INFO or CALL feature types

   (a) DP_DP - ratio of call depth over total variant depth (generally 1.0 for single-sample VCFs)

   (b) QUAL - the quality value in the VCF (QUAL)

   (c) NEARBY - the number of non-reference variant calls near the current variant (±20bp)

   (d) FILTER - the number of non-PASS filter values in the FILTER field of the VCF

   (e) ID - set to True (i.e. 1) if the ID field is not empty, otherwise False (i.e. 0)

3.2 Model Hyperparameters

During cross-validation, the models are given a selection of hyperparameters (i.e. parameters that define how the models are built) to choose from to identify the "best" combination of hyperparameters for the particular dataset. We selected a handful of hyperparameters based on the recommendations provided by `sklearn`, `imblearn`, and/or the corresponding literature for the models. We then applied `sklearn`'s `GridSearchSV` method which systematically tests every possible combination of hyperparameters of those provided. Only the best hyperparameters are then used for the final training and testing.

Table 2 reproduces the list of hyperparameters that were initially tested. Note that this list is not exhaustive, but is intended to represent the most impactful hyperparameters. Additionally, this list of hyperparameters is statically entered into this document, but it is subject to change with new versions and is best found embedded within the source code in file {`REPO`}`/scripts/TrainModels.py`.

Model	Hyperparameter	Search Space
RandomForestClassifier (sklearn)	random_state	[0]
	class_weight	['balanced']
	n_estimators	[100, 200]
	max_depth	[3, 4]
	min_samples_split	[2]
	max_features	['sqrt']
AdaBoostClassifier (sklearn)	random_state	[0]
	base_estimator	[DecisionTreeClassifier(max_depth=2)]
	n_estimators	[100, 200]
	learning_rate	[0.01, 0.1, 1.0]
	algorithm	['SAMME', 'SAMME.R']
GradientBoostingClassifier (sklearn)	random_state	[0]
	n_estimators	[100, 200]
	max_depth	[3, 4]
	learning_rate	[0.05, 0.1, 0.2]
	loss	['deviance', 'exponential']
	max_features	['sqrt']
EasyEnsembleClassifier (imblearn)	random_state	[0]
	n_estimators	[10, 20, 30, 40, 50]

Table 2: Hyperparameters tested in the initial version of the training pipeline.

3.3 Clinical Model Selection Formula

After full training, the models are evaluated on the unseen test dataset. Any candidate models are required to pass a cross-validation capture rate requirement and final capture rate requirement (see main document for details). We then use the following methodology to select the "best" candidate model that will ultimately

be used clinically. Note that this process is used for each variant/genotype combination, culminating in up to six final models (one per combo).

1. Let $S_m = 0.99$ be the minimum acceptable capture rate and $S_t = 0.995$ be the target capture rate for the models.
2. For each candidate model, let S be the final capture rate and F be the final TP flag rate for the model.
3. Calculate the scaled capture rate score, that is at most 1.0 (representing a model reaching the target capture rate): $S_s = min(1.0, \frac{S - S_m}{S_t - S_m})$
4. Calculate the machine learning specificity (true negative rate), $T = 1.0 - F$, such that higher values indicate fewer true variant calls being incorrectly sent for confirmation.
5. Calculate the modified F1 score: $F = harmonic\_mean(S_s, T)$
6. Of the remaining models, select the model with the highest F1 score, F, for use clinically.

Sample	True Positives	False Positives	Sensitivity	Precision	F-measure
SL362490 (NA12878)	3,526,846	17,164	0.9955	0.9952	0.9954
SL362491 (NA12878)	3,527,734	16,757	0.9958	0.9953	0.9955
SL362492 (NA12878)	3,530,085	17,184	0.9965	0.9952	0.9958
SL409548 (HG002)	3,485,975	22,200	0.9945	0.9937	0.9941
SL409549 (HG003)	3,336,817	20,287	0.9948	0.9940	0.9944
SL409550 (HG004)	3,368,531	30,167	0.9937	0.9911	0.9924
SL409551 (HG005)	3,298,988	13,388	0.9971	0.9960	0.9965
Mean±Stdev	3,439,282±93,430	19,592±5,033	0.9954±0.0011	0.9944±0.0015	0.9949±0.0013

Table 3: Summary metrics from RTG vcfeval for aligner "dragen-07.011.352.3.2.8b" and variant caller "dragen-07.011.352.3.2.8b".

4 Results for dragen-07.011.352.3.2.8b/dragen-07.011.352.3.2.8b

The following sections denote results that are specific the the pipeline consisting of aligner "dragen-07.011.352.3.2.8b" and variant caller "dragen-07.011.352.3.2.8b".

4.1 RTG vcfeval Results

The following sections contain results as reported by `rtg vcfeval`. For information on how `rtg vcfeval` was invoked, refer to Section 2.3.

4.1.1 Pipeline Performance

Table 3 contains the results from the RTG vcfeval `summary.txt` file that primarily contains summary information regarding the evaluated VCF file. We copied the results from this summary (unfiltered "None" row) and calculated summary mean and standard deviation as well.

Sensitivity is the fraction of annotated true positives that were correctly identified by the pipeline, precision is the fraction of called variants that were part of the truth set, and F-measure is the harmonic mean of sensitivity and precision. A perfect caller would have 1.0000 for all scores.

4.1.2 Variant Counts

Table 4 contains a summary of the number of false and true positive variant calls after stratifying the results by variant type and genotype.

4.2 Model Results

The following sections contain results specific to the final trained models.

4.2.1 Selected Models

Table 5 contains the selected models for aligner "dragen-07.011.352.3.2.8b" and caller "dragen-07.011.352.3.2.8b" given the minimum capture rate, $S_m = 0.99$, and the target capture rate, $S_t = 0.995$.

4.2.2 Strict Models

Table 6 contains the strict models for aligner "dragen-07.011.352.3.2.8b" and caller "dragen-07.011.352.3.2.8b" given the minimum capture rate, $S_m = 0.999$, and the target capture rate, $S_t = 1.0$. These models are labeled strict due to very high requirements, and the majority of models at different evaluation capture rates fail to pass these criteria. As a result, many variant/genotype combinations have no passing models or have models that are not practically useful (e.g. a TP flag rate of 99%).

Sample	RTG Result	SNV-HET	SNV-HOM	SNV-HE2	INDEL-HET	INDEL-HOM	INDEL-HE2	Total Calls
SL362490 (NA12878)	FP	3,133	543	5	9,351	1,913	2,217	17,162
SL362491 (NA12878)	FP	2,997	521	3	9,224	1,806	2,205	16,756
SL362492 (NA12878)	FP	3,372	484	8	9,734	1,260	2,325	17,183
SL409548 (HG002)	FP	5,527	956	8	11,589	1,536	2,584	22,200
SL409549 (HG003)	FP	4,583	721	8	10,933	1,424	2,617	20,286
SL409550 (HG004)	FP	6,018	770	5	16,898	2,156	4,319	30,166
SL409551 (HG005)	FP	4,553	712	8	5,870	1,208	1,036	13,387
Total	FP	30,183	4,707	45	73,599	11,303	17,303	137,140
SL362490 (NA12878)	TP	1,845,739	1,192,614	838	285,389	170,848	31,416	3,526,844
SL362491 (NA12878)	TP	1,845,930	1,192,707	835	285,750	170,952	31,559	3,527,733
SL362492 (NA12878)	TP	1,846,038	1,192,575	833	287,380	171,015	32,243	3,530,084
SL409548 (HG002)	TP	1,860,569	1,160,289	889	275,934	160,381	27,909	3,485,971
SL409549 (HG003)	TP	1,742,984	1,132,667	824	271,048	162,620	26,674	3,336,817
SL409550 (HG004)	TP	1,780,448	1,122,038	808	276,683	161,507	27,047	3,368,531
SL409551 (HG005)	TP	1,646,670	1,253,290	792	224,184	153,317	20,734	3,298,987
Total	TP	12,568,378	8,246,180	5,819	1,906,368	1,150,640	197,582	24,074,967

Table 4: This table shows the number of false and true positive variants calls as reported by `rtg vcfeval` for the aligner dragen-07.011.352.3.2.8b and variant caller dragen-07.011.352.3.2.8b. The variants are further divided by variant type (SNV or INDEL) and genotype (HET=heterozygous, HOM=homozygous, HE2=complex heterozygous). The "total" label refers to the sum of all samples for the corresponding "RTG Result" type.

Variant type	Best Model	Evaluation Capture Rate	CV Capture Rate	Final Capture Rate	CV TP flag rate	Final TP flag rate
SNV-HET	GradientBoosting	0.998	0.9976+-0.0018	0.9958	0.1278+-0.0226	0.1220
SNV-HOM	EasyEnsemble	0.9999	0.9994+-0.0014	0.9975	0.1725+-0.0207	0.1740
SNV-HE2	None	None	–	–	–	–
INDEL-HET	GradientBoosting	0.997	0.9962+-0.0026	0.9968	0.4311+-0.0335	0.4341
INDEL-HOM	GradientBoosting	0.998	0.9978+-0.0027	0.9950	0.5565+-0.0416	0.5516
INDEL-HE2	GradientBoosting	0.999	0.9986+-0.0014	0.9960	0.5345+-0.0565	0.5422

Table 5: Selected models for aligner "dragen-07.011.352.3.2.8b", caller "dragen-07.011.352.3.2.8b", $S_m = 0.99$, $S_t = 0.995$. If no model passed the criteria, then the "Best Model" field will be "None". Evaluation capture rate is the training capture that was used to gather results for the remaining fields in testing. Results prefaced with "CV" represent the test results during cross-validation. Similarly, results prefaced with "Final" represent the results on the held-out testing set during final evaluation. Note that we required the models to have capture requirements based on both the CV and Final results. In contrast, TP flag rate is not bound by any requirements, but is instead representative of the expected fraction of orthogonal confirmations required if the model is used.

Variant type	Best Model	Evaluation Capture Rate	CV Capture Rate	Final Capture Rate	CV TP flag rate	Final TP flag rate
SNV-HET	EasyEnsemble	1.0	0.9999+-0.0002	0.9999	0.8805+-0.0239	0.8885
SNV-HOM	None	None	–	–	–	–
SNV-HE2	None	None	–	–	–	–
INDEL-HET	GradientBoosting	0.9999	0.9999+-0.0002	0.9999	0.7957+-0.0536	0.8144
INDEL-HOM	AdaBoost	0.9999	0.9998+-0.0004	0.9995	0.8404+-0.0329	0.8414
INDEL-HE2	RandomForest	0.9999	0.9999+-0.0003	0.9998	0.9767+-0.0228	0.9867

Table 6: Strict models for aligner "dragen-07.011.352.3.2.8b", caller "dragen-07.011.352.3.2.8b", $S_m = 0.999$, $S_t = 1.0$. If no model passed the criteria, then the "Best Model" field will be "None". Evaluation capture rate is the training capture rate that was used to gather results for the remaining fields in testing. Results prefaced with "CV" represent the test results during cross-validation. Similarly, results prefaced with "Final" represent the results on the held-out testing set during final evaluation. Note that we required the models to have capture rate requirements based on both the CV and Final results. In contrast, TP flag rate is not bound by any requirements, but is instead representative of the expected fraction of orthogonal confirmations required if the model is used.

Feature	SNV-HET	SNV-HOM	SNV-HE2	INDEL-HET	INDEL-HOM	INDEL-HE2	Cumulative
CALL-GQ	0.3293	–	–	0.4679	0.2290	0.6180	1.6442
MUNGED-FILTER	0.1462	–	–	0.2199	0.0000	0.0002	0.3663
CALL-AD0	0.0265	–	–	0.0077	0.2243	0.0280	0.2866
CALL-AF1	0.0756	–	–	0.0968	0.0201	0.0811	0.2736
MUNGED-QUAL	0.0444	–	–	0.0759	0.0686	0.0720	0.2609
INFO-DB	0.1511	–	–	0.0338	0.0275	0.0184	0.2308
CALL-DP	0.0111	–	–	0.0034	0.1712	0.0185	0.2041
CALL-AD1	0.0162	–	–	0.0239	0.1164	0.0461	0.2025
MUNGED-NEARBY	0.0688	–	–	0.0156	0.0444	0.0079	0.1367
CALL-AF0	0.0173	–	–	0.0201	0.0234	0.0660	0.1268
INFO-FractionInformativeReads	0.0236	–	–	0.0062	0.0351	0.0069	0.0718
INFO-MQ	0.0374	–	–	0.0071	0.0148	0.0074	0.0667
INFO-MQRankSum	0.0224	–	–	0.0128	0.0069	0.0142	0.0563
MUNGED-DP_DP	0.0207	–	–	0.0050	0.0108	0.0071	0.0436
INFO-ReadPosRankSum	0.0077	–	–	0.0039	0.0047	0.0056	0.0218
INFO-R2_5P_bias	0.0018	–	–	0.0002	0.0028	0.0024	0.0072
INFO-QD	0.0000	–	–	0.0000	0.0000	0.0000	0.0000
INFO-FS	0.0000	–	–	0.0000	0.0000	0.0000	0.0000
INFO-SOR	0.0000	–	–	0.0000	0.0000	0.0000	0.0000

Table 7: This table shows the feature importances results for aligner "dragen-07.011.352.3.2.8b" and caller "dragen-07.011.352.3.2.8b". Importances are broken down by category with a cumulative sum at the end. Note that some results may be missing if the pipeline was run incorrectly or the models are not interpretable through `eli5`.

4.2.3 Feature Importances

Table 7 contains results regarding feature importances according to the models. These were gathered using the `eli5` package and the `ExtractELI5Results.py` script from this repo. Feature importances may be missing due to any of the following reasons:

1. ELI5 interpretation was not run correctly - This could be because the `ExtractELI5Results.py` script has not been executed or the outputs are not in the expected location.
2. The model failed to pass our base clinical criteria - We restricted the outputs to only include models that met the minimum capture rate requirement as defined in the "Selected Models" section above.
3. The model is not interpretable by `eli5` - Not all models provide feature importance measures through eli5 so these results are excluded

4.2.4 Model for SNV-HET

Figure 1 contains the receiver-operator curves (ROC) for the final trained models for aligner "dragen-07.011.352.3.2.8b", caller "dragen-07.011.352.3.2.8b", variant type "SNV", and genotype "HET".

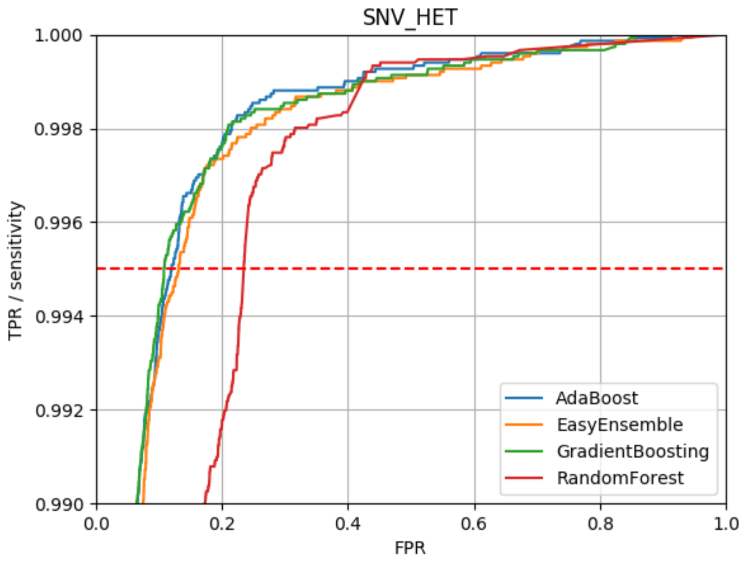

Figure 1: ROC curve for aligner “dragen-07.011.352.3.2.8b”, caller “dragen-07.011.352.3.2.8b”, variant type “SNV”, and genotype “HET”. Note that these curves are zoomed in to focus on only the region greater than the minimum clinical capture rate (0.99).

4.2.5 Model for SNV-HOM

Figure 2 contains the receiver-operator curves (ROC) for the final trained models for aligner “dragen-07.011.352.3.2.8b”, caller “dragen-07.011.352.3.2.8b”, variant type “SNV”, and genotype “HOM”.

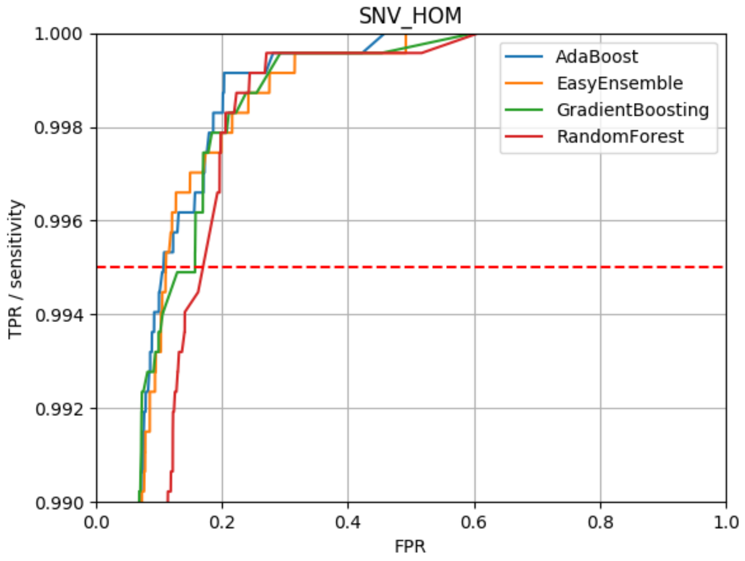

Figure 2: ROC curve for aligner “dragen-07.011.352.3.2.8b”, caller “dragen-07.011.352.3.2.8b”, variant type “SNV”, and genotype “HOM”. Note that these curves are zoomed in to focus on only the region greater than the minimum clinical capture rate (0.99).

4.2.6 Model for SNV-HE2

Figure 3 contains the receiver-operator curves (ROC) for the final trained models for aligner "dragen-07.011.352.3.2.8b", caller "dragen-07.011.352.3.2.8b", variant type "SNV", and genotype "HE2".

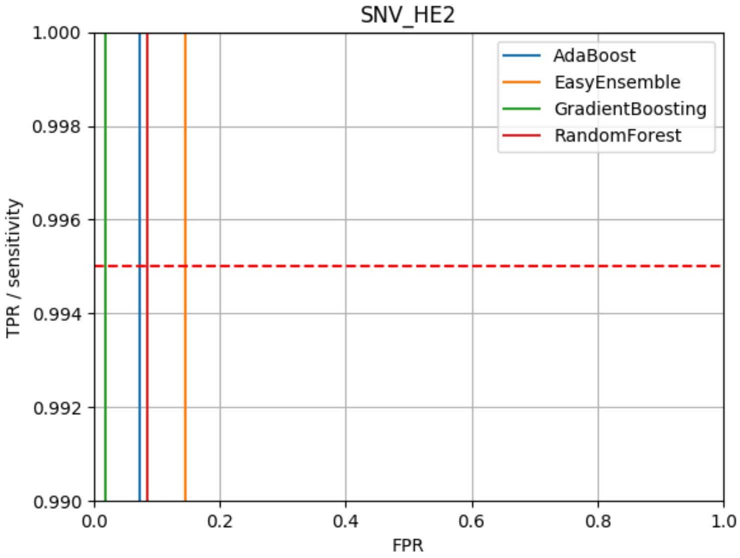

Figure 3: ROC curve for aligner "dragen-07.011.352.3.2.8b", caller "dragen-07.011.352.3.2.8b", variant type "SNV", and genotype "HE2". Note that these curves are zoomed in to focus on only the region greater than the minimum clinical capture rate (0.99).

4.2.7 Model for INDEL-HET

Figure 4 contains the receiver-operator curves (ROC) for the final trained models for aligner "dragen-07.011.352.3.2.8b", caller "dragen-07.011.352.3.2.8b", variant type "INDEL", and genotype "HET".

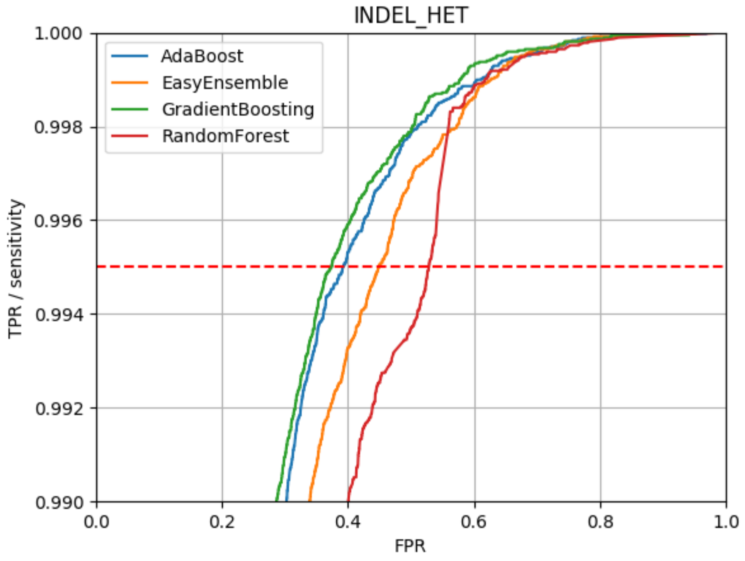

Figure 4: ROC curve for aligner "dragen-07.011.352.3.2.8b", caller "dragen-07.011.352.3.2.8b", variant type "INDEL", and genotype "HET". Note that these curves are zoomed in to focus on only the region greater than the minimum clinical capture rate (0.99).

4.2.8 Model for INDEL-HOM

Figure 5 contains the receiver-operator curves (ROC) for the final trained models for aligner "dragen-07.011.352.3.2.8b", caller "dragen-07.011.352.3.2.8b", variant type "INDEL", and genotype "HOM".

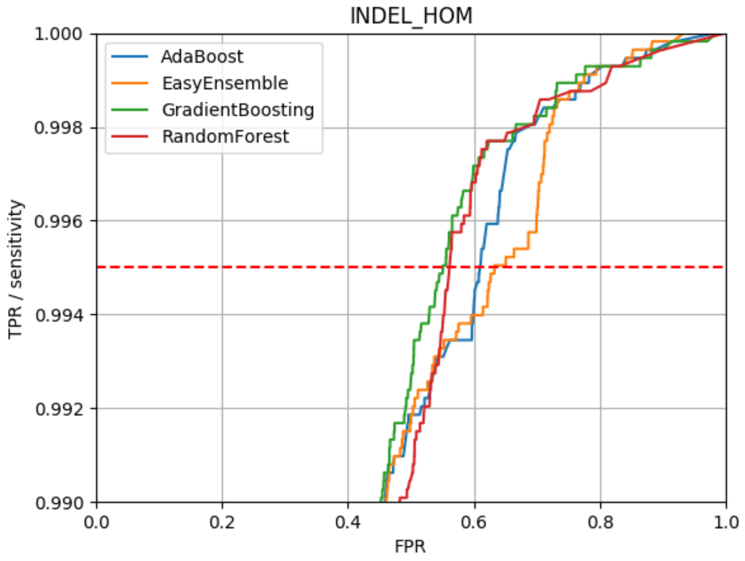

Figure 5: ROC curve for aligner "dragen-07.011.352.3.2.8b", caller "dragen-07.011.352.3.2.8b", variant type "INDEL", and genotype "HOM". Note that these curves are zoomed in to focus on only the region greater than the minimum clinical capture rate (0.99).

4.2.9 Model for INDEL-HE2

Figure 6 contains the receiver-operator curves (ROC) for the final trained models for aligner "dragen-07.011.352.3.2.8b", caller "dragen-07.011.352.3.2.8b", variant type "INDEL", and genotype "HE2".

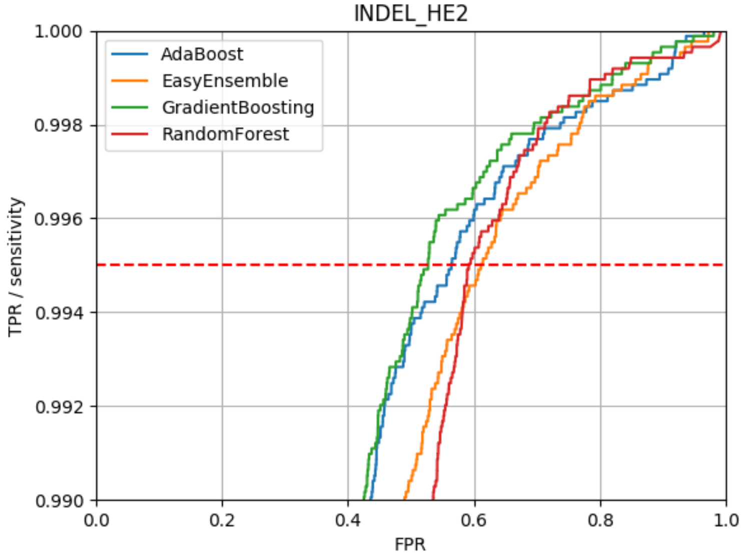

Figure 6: ROC curve for aligner "dragen-07.011.352.3.2.8b", caller "dragen-07.011.352.3.2.8b", variant type "INDEL", and genotype "HE2". Note that these curves are zoomed in to focus on only the region greater than the minimum clinical capture rate (0.99).

Sample	True Positives	False Positives	Sensitivity	Precision	F-measure
SL362490 (NA12878)	3,525,034	54,412	0.9948	0.9848	0.9898
SL362491 (NA12878)	3,526,087	51,499	0.9951	0.9856	0.9903
SL362492 (NA12878)	3,530,183	48,285	0.9962	0.9865	0.9913
SL409548 (HG002)	3,487,420	66,187	0.9947	0.9814	0.9880
SL409549 (HG003)	3,338,699	61,468	0.9951	0.9819	0.9884
SL409550 (HG004)	3,366,577	88,137	0.9928	0.9745	0.9836
SL409551 (HG005)	3,296,993	49,769	0.9963	0.9851	0.9907
Mean±Stdev	3,438,713±93,439	59,965±12,970	0.9950±0.0011	0.9828±0.0038	0.9889±0.0024

Table 8: Summary metrics from RTG vcfeval for aligner "sentieon-201808.07" and variant caller "strelka-2.9.10".

5 Results for sentieon-201808.07/strelka-2.9.10

The following sections denote results that are specific the the pipeline consisting of aligner "sentieon-201808.07" and variant caller "strelka-2.9.10".

5.1 RTG vcfeval Results

The following sections contain results as reported by `rtg vcfeval`. For information on how `rtg vcfeval` was invoked, refer to Section 2.3.

5.1.1 Pipeline Performance

Table 8 contains the results from the RTG vcfeval `summary.txt` file that primarily contains summary information regarding the evaluated VCF file. We copied the results from this summary (unfiltered "None" row) and calculated summary mean and standard deviation as well.

Sensitivity is the fraction of annotated true positives that were correctly identified by the pipeline, precision is the fraction of called variants that were part of the truth set, and F-measure is the harmonic mean of sensitivity and precision. A perfect caller would have 1.0000 for all scores.

5.1.2 Variant Counts

Table 9 contains a summary of the number of false and true positive variant calls after stratifying the results by variant type and genotype.

5.2 Model Results

The following sections contain results specific to the final trained models.

5.2.1 Selected Models

Table 10 contains the selected models for aligner "sentieon-201808.07" and caller "strelka-2.9.10" given the minimum capture rate, $S_m = 0.99$, and the target capture rate, $S_t = 0.995$.

5.2.2 Strict Models

Table 11 contains the strict models for aligner "sentieon-201808.07" and caller "strelka-2.9.10" given the minimum capture rate, $S_m = 0.999$, and the target capture rate, $S_t = 1.0$. These models are labeled strict due to very high requirements, and the majority of models at different evaluation capture rates fail to pass these criteria. As a result, many variant/genotype combinations have no passing models or have models that are not practically useful (e.g. a TP flag rate of 99%).

Sample	RTG Result	SNV-HET	SNV-HOM	SNV-HE2	INDEL-HET	INDEL-HOM	INDEL-HE2	Total Calls
SL362490 (NA12878)	FP	19,475	316	13	28,467	4,681	1,369	54,321
SL362491 (NA12878)	FP	16,960	261	9	28,009	4,781	1,345	51,365
SL362492 (NA12878)	FP	13,941	200	17	28,423	4,117	1,370	48,068
SL409548 (HG002)	FP	24,560	434	16	35,297	4,239	1,635	66,181
SL409549 (HG003)	FP	19,935	396	11	35,550	3,966	1,680	61,538
SL409550 (HG004)	FP	37,516	343	25	41,365	6,695	2,155	88,099
SL409551 (HG005)	FP	27,772	278	10	18,150	2,931	683	49,824
Total	FP	160,159	2,228	101	215,261	31,410	10,237	419,396
SL362490 (NA12878)	TP	1,848,561	1,192,431	837	283,154	171,678	28,315	3,524,976
SL362491 (NA12878)	TP	1,848,751	1,192,465	840	283,699	171,887	28,395	3,526,037
SL362492 (NA12878)	TP	1,849,072	1,192,443	841	285,800	172,349	29,655	3,530,160
SL409548 (HG002)	TP	1,863,668	1,159,651	890	273,971	161,656	25,104	3,484,940
SL409549 (HG003)	TP	1,745,901	1,132,245	825	269,263	163,809	24,177	3,336,220
SL409550 (HG004)	TP	1,783,155	1,121,260	812	273,794	163,741	23,550	3,366,312
SL409551 (HG005)	TP	1,648,579	1,252,589	793	222,881	153,310	18,756	3,296,908
Total	TP	12,587,687	8,243,084	5,838	1,892,562	1,158,430	177,952	24,065,553

Table 9: This table shows the number of false and true positive variants calls as reported by `rtg vcfeval` for the aligner sentieon-201808.07 and variant caller strelka-2.9.10. The variants are further divided by variant type (SNV or INDEL) and genotype (HET=heterozygous, HOM=homozygous, HE2=complex heterozygous). The "total" label refers to the sum of all samples for the corresponding "RTG Result" type.

Variant type	Best Model	Evaluation Capture Rate	CV Capture Rate	Final Capture Rate	CV TP flag rate	Final TP flag rate
SNV-HET	GradientBoosting	0.996	0.9958+-0.0007	0.9952	0.0166+-0.0026	0.0167
SNV-HOM	EasyEnsemble	0.998	0.9987+-0.0032	0.9955	0.1543+-0.0566	0.1483
SNV-HE2	None	None	–	–	–	–
INDEL-HET	GradientBoosting	0.996	0.9958+-0.0011	0.9950	0.2040+-0.0328	0.2029
INDEL-HOM	GradientBoosting	0.997	0.9968+-0.0015	0.9955	0.4235+-0.0398	0.4243
INDEL-HE2	GradientBoosting	0.997	0.9965+-0.0019	0.9955	0.6501+-0.0463	0.6495

Table 10: Selected models for aligner "sentieon-201808.07", caller "strelka-2.9.10", $S_m = 0.99$, $S_t = 0.995$. If no model passed the criteria, then the "Best Model" field will be "None". Evaluation capture rate is the training capture that was used to gather results for the remaining fields in testing. Results prefaced with "CV" represent the test results during cross-validation. Similarly, results prefaced with "Final" represent the results on the held-out testing set during final evaluation. Note that we required the models to have capture requirements based on both the CV and Final results. In contrast, TP flag rate is not bound by any requirements, but is instead representative of the expected fraction of orthogonal confirmations required if the model is used.

Variant type	Best Model	Evaluation Capture Rate	CV Capture Rate	Final Capture Rate	CV TP flag rate	Final TP flag rate
SNV-HET	GradientBoosting	0.9999	0.9999+-0.0002	0.9997	0.2863+-0.0344	0.2802
SNV-HOM	RandomForest	0.999	1.0000+-0.0000	1.0000	0.9973+-0.0021	0.9972
SNV-HE2	None	None	–	–	–	–
INDEL-HET	GradientBoosting	0.9999	0.9999+-0.0002	0.9997	0.6160+-0.0492	0.6192
INDEL-HOM	EasyEnsemble	0.9999	0.9999+-0.0004	0.9997	0.9258+-0.0200	0.9296
INDEL-HE2	None	None	–	–	–	–

Table 11: Strict models for aligner "sentieon-201808.07", caller "strelka-2.9.10", $S_m = 0.999$, $S_t = 1.0$. If no model passed the criteria, then the "Best Model" field will be "None". Evaluation capture rate is the training capture rate that was used to gather results for the remaining fields in testing. Results prefaced with "CV" represent the test results during cross-validation. Similarly, results prefaced with "Final" represent the results on the held-out testing set during final evaluation. Note that we required the models to have capture rate requirements based on both the CV and Final results. In contrast, TP flag rate is not bound by any requirements, but is instead representative of the expected fraction of orthogonal confirmations required if the model is used.

5.2.3 Feature Importances

Table 12 contains results regarding feature importances according to the models. These were gathered using the `eli5` package and the `ExtractELI5Results.py` script from this repo. Feature importances may be missing due to any of the following reasons:

1. ELI5 interpretation was not run correctly - This could be because the `ExtractELI5Results.py` script has not been executed or the outputs are not in the expected location.
2. The model failed to pass our base clinical criteria - We restricted the outputs to only include models that met the minimum capture rate requirement as defined in the "Selected Models" section above.
3. The model is not interpretable by `eli5` - Not all models provide feature importance measures through eli5 so these results are excluded

5.2.4 Model for SNV-HET

Figure 7 contains the receiver-operator curves (ROC) for the final trained models for aligner "sentieon-201808.07", caller "strelka-2.9.10", variant type "SNV", and genotype "HET".

Feature	SNV-HET	SNV-HOM	SNV-HE2	INDEL-HET	INDEL-HOM	INDEL-HE2	Cumulative
CALL-GQX	0.1562	–	–	0.3838	0.4500	0.3621	1.3521
MUNGED-QUAL	0.0122	–	–	0.4038	0.2845	0.0842	0.7847
MUNGED-FILTER	0.2982	–	–	0.0955	0.0158	0.1190	0.5284
CALL-GQ	0.0463	–	–	0.0219	0.1071	0.1170	0.2923
CALL-SB	0.2840	–	–	0.0000	0.0000	0.0000	0.2840
CALL-AF1	0.0299	–	–	0.0337	0.0232	0.1407	0.2275
CALL-AD1	0.1194	–	–	0.0098	0.0208	0.0263	0.1763
CALL-AF0	0.0040	–	–	0.0104	0.0434	0.0555	0.1134
MUNGED-ID	0.0377	–	–	0.0208	0.0068	0.0118	0.0771
CALL-AD0	0.0025	–	–	0.0077	0.0347	0.0272	0.0722
CALL-DPI	0.0000	–	–	0.0059	0.0062	0.0340	0.0460
INFO-MQ	0.0014	–	–	0.0043	0.0032	0.0140	0.0230
MUNGED-NEARBY	0.0029	–	–	0.0023	0.0043	0.0083	0.0178
INFO-SNVHPOL	0.0037	–	–	0.0000	0.0000	0.0000	0.0037
CALL-DP	0.0012	–	–	0.0000	0.0000	0.0000	0.0012
CALL-DPF	0.0004	–	–	0.0000	0.0000	0.0000	0.0004

Table 12: This table shows the feature importances results for aligner "sentieon-201808.07" and caller "strelka-2.9.10". Importances are broken down by category with a cumulative sum at the end. Note that some results may be missing if the pipeline was run incorrectly or the models are not interpretable through `eli5`.

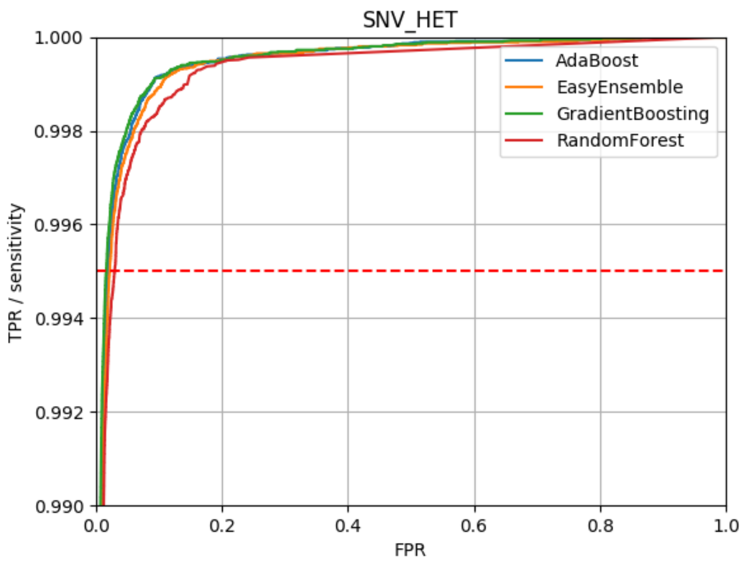

Figure 7: ROC curve for aligner "sentieon-201808.07", caller "strelka-2.9.10", variant type "SNV", and genotype "HET". Note that these curves are zoomed in to focus on only the region greater than the minimum clinical capture rate (0.99).

5.2.5 Model for SNV-HOM

Figure 8 contains the receiver-operator curves (ROC) for the final trained models for aligner "sentieon-201808.07", caller "strelka-2.9.10", variant type "SNV", and genotype "HOM".

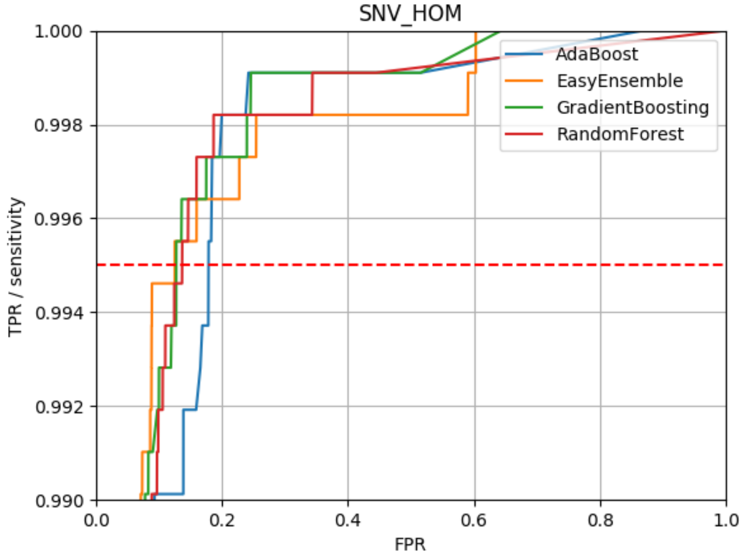

Figure 8: ROC curve for aligner "sentieon-201808.07", caller "strelka-2.9.10", variant type "SNV", and genotype "HOM". Note that these curves are zoomed in to focus on only the region greater than the minimum clinical capture rate (0.99).

5.2.6 Model for SNV-HE2

Figure 9 contains the receiver-operator curves (ROC) for the final trained models for aligner "sentieon-201808.07", caller "strelka-2.9.10", variant type "SNV", and genotype "HE2".

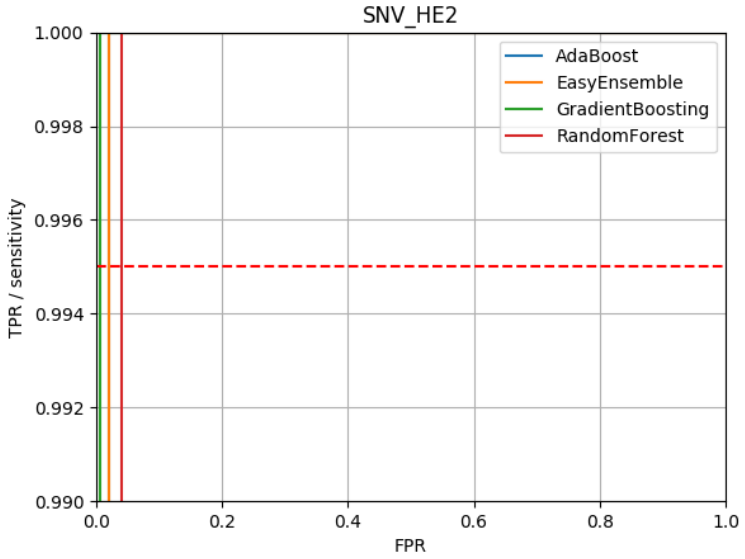

Figure 9: ROC curve for aligner "sentieon-201808.07", caller "strelka-2.9.10", variant type "SNV", and genotype "HE2". Note that these curves are zoomed in to focus on only the region greater than the minimum clinical capture rate (0.99).

5.2.7 Model for INDEL-HET

Figure 10 contains the receiver-operator curves (ROC) for the final trained models for aligner "sentieon-201808.07", caller "strelka-2.9.10", variant type "INDEL", and genotype "HET".

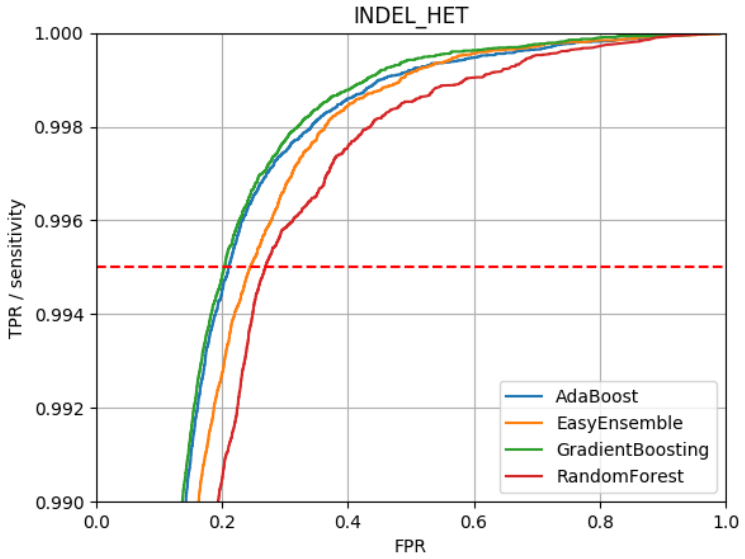

Figure 10: ROC curve for aligner "sentieon-201808.07", caller "strelka-2.9.10", variant type "INDEL", and genotype "HET". Note that these curves are zoomed in to focus on only the region greater than the minimum clinical capture rate (0.99).

5.2.8 Model for INDEL-HOM

Figure 11 contains the receiver-operator curves (ROC) for the final trained models for aligner "sentieon-201808.07", caller "strelka-2.9.10", variant type "INDEL", and genotype "HOM".

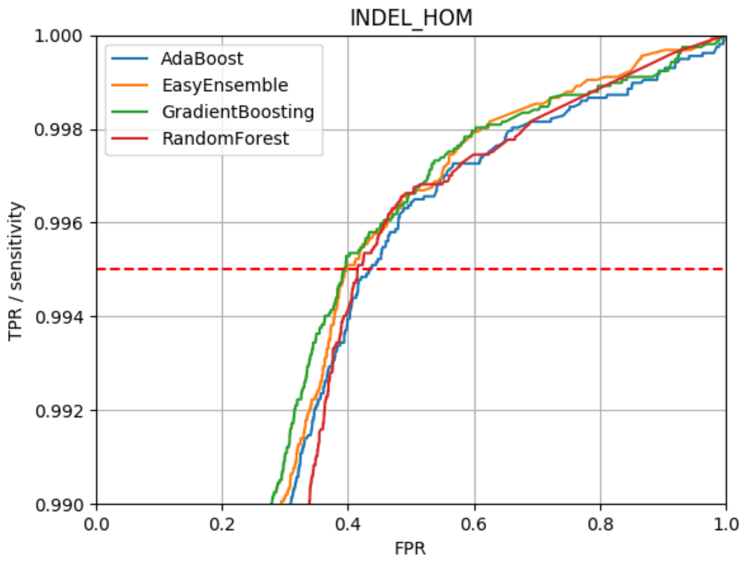

Figure 11: ROC curve for aligner "sentieon-201808.07", caller "strelka-2.9.10", variant type "INDEL", and genotype "HOM". Note that these curves are zoomed in to focus on only the region greater than the minimum clinical capture rate (0.99).

5.2.9 Model for INDEL-HE2

Figure 12 contains the receiver-operator curves (ROC) for the final trained models for aligner "sentieon-201808.07", caller "strelka-2.9.10", variant type "INDEL", and genotype "HE2".

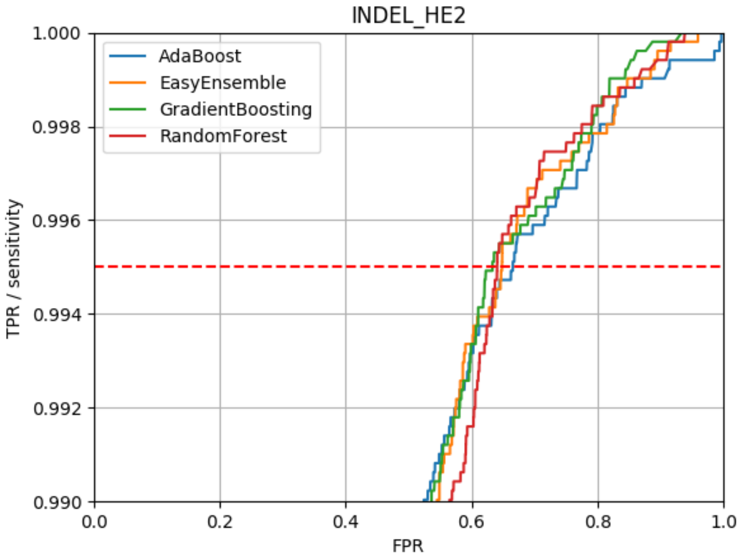

Figure 12: ROC curve for aligner "sentieon-201808.07", caller "strelka-2.9.10", variant type "INDEL", and genotype "HE2". Note that these curves are zoomed in to focus on only the region greater than the minimum clinical capture rate (0.99).

6 HG006 and HG007 Experiment

The following sections outline data from experiments using samples HG006 and HG007. The data presented here is hard-coded into the document and currently is not auto-generated from the training process.

6.1 Datasets

Table 13 contains the metadata for two samples (HG006 and HG007). These samples were not used for training, but only used to test the fully trained clinical models.

Library	Sample	Preparation	Mean Coverage
SL455110	HG006	Clinical PCR	35.98
SL455111	HG007	Clinical PCR	33.69

Table 13: This table contains metadata regarding the two follow-up samples that were not used the train the clinical models.

6.2 RTG vcfeval Performance

Due to updates to the Dragen software, we were not able to run the above samples on the exact same version of Dragen as the training samples. Instead, they were run using Amazon AWS Dragen software version 05.021.510.3.5.7 (version 3.5.7).

To focus the analysis, we restricted the regions that were analyzed to the intersection between the benchmark regions and our set of annotated exon regions. This substantially reduced the total number of variants per sample, but also removed many of the unusual regions we identified during analysis (see next section for details). Table 14 contains the results from the RTG vcfeval `summary.txt` file that contains summary information regarding the evaluated VCF file.

Sensitivity is the fraction of annotated true positives that were correctly identified by the pipeline, precision is the fraction of called variants that were part of the truth set, and F-measure is the harmonic mean of sensitivity and precision. A perfect caller would equal 1.0000 for all scores.

Sample	True Positives	False Positives	Sensitivity	Precision	F-measure
SL455110 (HG006-full)	3378563	13144	0.9971	0.9961	0.9966
SL455111 (HG007-full)	3397333	13823	0.9968	0.9959	0.9964
SL455110 (HG006-exons)	103285	151	0.9990	0.9985	0.9988
SL455111 (HG007-exons)	104054	188	0.9989	0.9982	0.9985

Table 14: Summary metrics from RTG vcfeval for aligner “dragen-05.021.510.3.5.7” and variant caller “dragen-05.021.510.3.5.7”.

6.3 Observations from the HG006 and HG007 experiments

6.3.1 Background

Our training pipeline used HG001-005 to perform a leave-one-sample-out cross-validation. With this completed, two samples that were not involved in model training, HG006 and HG007 GIAB samples, were tested with the trained models. These two samples were sequenced about 1 year after sequencing the samples used to train the model using our standard clinical workflow. These data passed our quality control evaluation used for clinical sequencing data.

These two samples were run through our sentieon-strelka2 pipeline in a manner identical to the training samples. Unfortunately, the exact Dragen version used for training was not available due to upgrades. Therefore, we loaded a Dragen instance running v3.5.7 on Amazon Web Services to stand up a Dragen instance running v3.5.7. It should be noted that the models were trained on v3.2.8. When the outputs of both pipelines were compared to the benchmark regions, their performance was comparable to the previous samples (HG001-HG005) in terms of recall and precision (see Table 13).

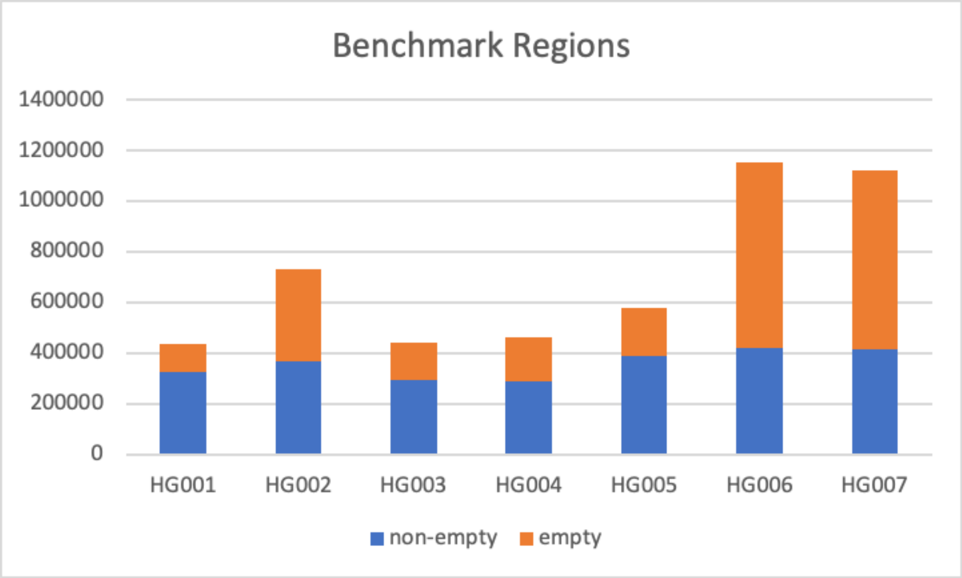

Figure 13: The total number of benchmark regions per GIAB sample. These are split into empty (no variants) and non-empty (at least one variant) regions.

We then used the variants from HG006 and HG007 to test the models that were trained on HG001-HG005. For both samples and both pipelines, the results were poorer than expected with most of the models achieving less than 99% capture rate for the false positive calls. This was an unexpected result, as testing a similar approach by leaving HG005 out of the training data had yielded a satisfactory result. After a series of debugging steps that included adding HG006 to our training set, we were not able to improve the results on these datasets. In all of our experiments, any test where HG006 or HG007 was the unseen test data ended poorly (when we trained with HG006, this was noticeable in our cross-validation as well).

We considered the possibility that HG001-HG005 GIAB benchmarks were fundamentally different from HG006 and HG007 GIAB benchmarks. The following subsections detail some of our observations.

6.3.2 Different Processing Date

The GIAB FTP README file notes that the samples HG006 and HG007 were processed separately and at a later date than the other samples (HG001-HG005) for release 3.3.2.

6.3.3 Benchmark Regions

The benchmark regions for GIAB are stored in BED files consisting of a chromosome, a start coordinate, and an end coordinate for each region. First, the total number of benchmark regions for HG006 and HG007 is much larger than any other GIAB sample. Each one was almost double the count of HG005. Note that this trend is the *opposite* of the Ashkenazi Jewish trio (HG002-HG004), where both parents had fewer benchmark regions than the child. Second, there were far more "empty" regions in HG006 and HG007 (regions that were included in the benchmark with no variants in them). Over 60% of the regions are empty in HG006/HG007 (parents of Chinese trio) contrasted with about 35% in HG003/HG004 (parents of Ashkenazi trio). HG006 and HG007 both also have far more total empty regions than any other sample from GIAB, each having nearly twice the number of empty regions compared to the sample with the next highest number of empty regions (HG002). The total number of regions for each sample by empty and non-empty are shown in Figure 13.

6.3.4 Clustered False Positives

When we analyzed the false positive variants that were not captured by the trained models, they were more often "clustered" sequentially in the HG006 and HG007 datasets. While the variants in our dataset are stripped of identifying information (such as chromosome or position), they are still added sequentially to the feature set in the same order as the VCF. This means that these "clusters" of uncaptured false positive were more likely to be located near each other in genomic coordinate space, and they are also more likely to be within the same benchmark region. This cluster analysis is not entirely reliable for two reasons: 1) the false positives, while ordered by position, are not guaranteed to be within the same benchmark region and 2) the false positives of different variant types have been split into different subsets and therefore they are no longer adjacent in our feature matrix).

To further analyze this observation, we collected a list of clusters of false positive variants (at least 2 variants in a row) that were not captured by our models. This analysis was performed on the full list of false positives for HG006 and HG007 which includes both exonic and non-exonic benchmark regions. We then counted the total number of clusters, the average cluster length, and the maximum cluster length for each sample. In both of our pipelines, there were more clusters, a longer average cluster length, and a longer maximum cluster length for HG006 and HG007 compared to all the other samples. Our HG006 sample seemed to have more and longer clusters than the HG007 sample. Additionally, this effect is more obvious in the sentieon-strelka2 pipeline where there are more total false positive calls in the datasets. These measures are presented in Tables 15 and 16.

Sample	Total Clusters	Average Cluster Length	Max Cluster Length
SL362490 (HG001)	0	–	–
SL362491 (HG001)	2	2.0	2
SL362492 (HG001)	2	2.0	2
SL409548 (HG002)	1	2.0	2
SL409549 (HG003)	0	–	–
SL409550 (HG004)	0	–	–
SL409551 (HG005)	0	–	–
SL455110 (HG006)	31	2.74	8
SL455111 (HG007)	4	2.25	3

Table 15: Statistics on clusters of false positive variants that were not correctly captured by the machine learning models for aligner "dragen-05.021.510.3.5.7" and variant caller "dragen-05.021.510.3.5.7".

Sample	Total Clusters	Average Cluster Length	Max Cluster Length
SL362490 (HG001)	17	2.2	3
SL362491 (HG001)	15	2.3	5
SL362492 (HG001)	11	2.2	3
SL409548 (HG002)	12	2.0	2
SL409549 (HG003)	10	2.3	5
SL409550 (HG004)	12	2.1	3
SL409551 (HG005)	12	2.2	4
SL455110 (HG006)	83	2.5	12
SL455111 (HG007)	32	2.3	5

Table 16: Statistics on clusters of false positive variants that were not correctly captured by the machine learning models for aligner "sentieon-201808.07" and variant caller "strelka-2.9.10".

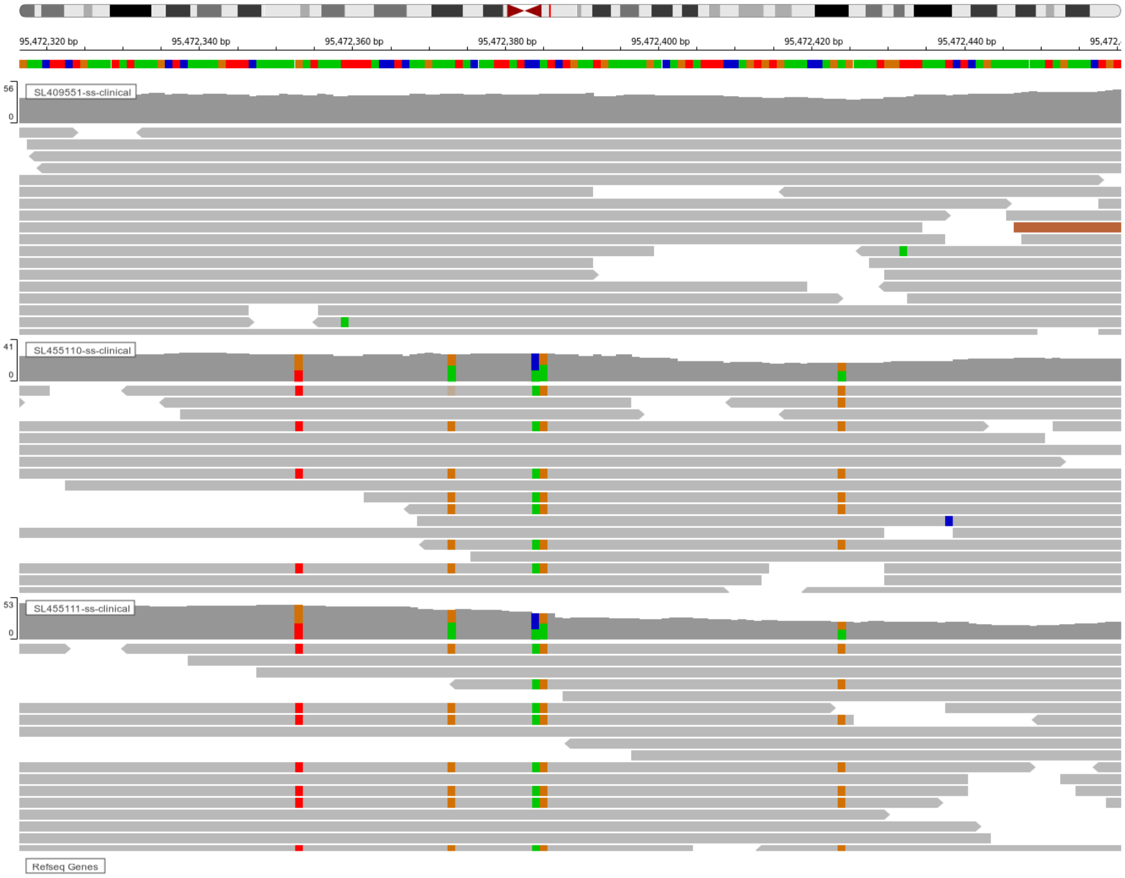

Figure 14: A manually inspected region where a run of variants were all labeled as missed false positives. These 5 heterozygous SNV variants visually appear real in HG006 (middle) and HG007 (bottom) but are visually absent in HG005 (top).

6.3.5 Manual inspection of one uncaptured run region

Visual inspection of one uncaptured region using the Integrative Genomic Viewer (IGV) provides a window into the "clustering" of uncaptured false positives. Coordinates are not included in the features for our variants, but by examining multiple features for the variants in one of these clusters, we were able to identify the variants in the false positive VCF file. They were all co-located in a single benchmark region (hg38, chr3:95472320-95472473). There are no variants in this benchmark region in the benchmark VCFs for HG005-HG007. However, in our variant calls for HG006 and HG007, both of our pipelines (Dragen and Sentieon/Strelka2) called 5 heterozygous SNVs. Visual inspection (via IGV) reveals that these variants are all on the same haplotype in both samples. These variants were not detected in HG005 (the child of HG006 and HG007), which would be consistent with inheriting the copies without the SNVs. Each of these five variants has a dbSNP identifier, and two of them reside in a repeat region according to the RepeatMasker track on UCSC. The alignments for all three samples appear to be relatively "clean", as in there are no obvious alignment artifacts present that would likely confound a variant caller. An IGV image of this region is in Figure 14.

While these variants were absent from the HG006 final VCF file, *all* of them appeared in a supplementary VCF labeled as "testing" for HG006.[1] They were labeled with the flag "discordantunfiltered" which has the description "Callsets with unfiltered calls have discordant genotypes or variant calls". Additionally, it was

[1]`https://ftp-trace.ncbi.nlm.nih.gov/giab/ftp/release/ChineseTrio/HG006_NA24694_father/NISTv3.3.2/GRCh38/supplementaryFiles/HG006_GIAB_GRCh38_highconf_CG-IllFB-IllSNT-10X_testing_CHROM1-22_v.3.3.2_all.vcf.gz`

noted that each was run on 3 platforms and was called with the heterozygous genotype in 5 pipelines used to generate the GIAB benchmark. Manual inspection of the region in the 6 files provided in the supplementary files input VCFs[2] confirms this, with only the file labeled `HG006_1-22_GRCh38_novoalign_Hiseq100X_FB.vcf.gz` missing the variant calls. Finally, the phase of the variants was established. 4 of the 5 variants are present on the same haplotype with the last one unphased according to the VCF file. We were unable to locate a similar "testing" VCF for HG007 to check for a similar phenomenon.

Our variant calls are consistent with the calls from the "testing" file (and 5 of the 6 input files), but none of those calls appear in the high-confidence call set. It appears that calls are removed (filtered out) when there is discordance among the 6 input files. Nevertheless, it is unclear why the region we examined is present in the benchmark BED file given that there are 5 discordant calls within the relatively small region of the chromosome. While we only inspected one region, we suspect similar phenomena are occurring in many of the additional, "empty" benchmark regions in HG006 and HG007.

6.3.6 A possible explanation of our findings

It appears that the process to generate the truth sets for HG006 and HG007 was different from the process(es) for HG001-HG005. We think the different processing date and the major difference in total number of benchmark regions are the clearest evidence supporting this possibility. After the targeted manual inspection, we further suspect that either: (1) some regions were erroneously added to the BED file, (2) that the variants were erroneously removed from the VCF file, or (3) a combination of the two. Given the excess number of benchmark regions, we believe that at least (1) is likely.

While this processing difference has a relatively minor influence on recall/precision for comparing aligners and variant callers (see our RTG results), we think this difference is a significant confounding factor for our problem of identifying false positives via machine learning. In particular, our observations suggest that many true variants are being incorrectly classified as false positives. This would manifest as reduced capture rates in our tests for HG006 and HG007 because the models would *correctly* recognize them as true positives, but that classification wouldn't match the result from RTG vcfeval. Given the above observations, we caution other researchers about the use v3.3.2 of HG006 and HG007 for the purpose of capturing false positives until their benchmark regions can be reconciled to the process used for HG001-HG005.

6.3.7 Addressing the issues with HG006 and HG007

It is unclear how to remove or correct the aforementioned phenomenon globally in HG006 and HG007. Removing all "empty" regions from the benchmark dataset did improve our results, but not enough to capture false positives at the same level as HG001-HG005. Assuming our data issue hypothesis is correct, this would suggest that the processing error is not confined to "empty" regions from the BED file.

We were able to control for the issue by reducing the GIAB benchmark regions to those that overlap exons (i.e. clinically relevant regions). This reduced the number of missed (uncaptured) false positive calls to 8 across both HG006 and HG007. This was a relatively manageable set of variants, so each was traced back to the original variant call by reviewing the features by hand and then verifying the absence from the truth set. We then evaluated all eight of these variants using two orthogonal methods: Sanger sequencing and PacBio HiFi sequencing. Detailed analysis of the 8 variants are presented in the next section.

6.4 Additional False Positive Information

After reducing the GIAB benchmark regions to those overlapping exons, we were left with 8 uncaptured, false positive calls across both HG006 and HG007. For each of these variants, we ordered orthogonal confirmation via Sanger sequencing. We also analyzed PacBio HiFi sequencing from HudsonAlpha that is

[2]https://ftp-trace.ncbi.nlm.nih.gov/giab/ftp/release/ChineseTrio/HG006_NA24694_father/NISTv3.3.2/GRCh38/supplementaryFiles/inputvcfsandbeds/

publicly available[3] [4] using three different aligners (minimap2-2.17[5], pbmm2-1.2.1[6], and sentieon-201808.07) followed by DeepVariant-1.1.0[7]. Where possible, we also investigated the GIAB HG006 "testing" file and corresponding input supplementary files (both HG006 and HG007) available from GIAB. The results of the Sanger tests, PacBio tests, and notes from our investigations are summarized in Table 17.

Of the 8 initial variants, we labeled 5 of them as "Confirmed TP", meaning that the confirmatory and supporting evidence points to them being incorrectly excluded from the truth set. The two variants from HG007 were labeled as "Likely TP" as they were found via Sanger or PacBio sequencing, but not both. The first one (chr16:68157911A>G) was captured via Sanger sequencing, but was not detected by the PacBio sequencing. The second one (chr11:56700783C>T) failed Sanger sequencing. For this variant, all of the PacBio HiFi pipelines reported a homozgyous variant at this locus. We labeled this as a "Likely TP" because all sources are in agreement that the variant is present, yet there is a disagreement regarding the zygosity of the genotype call. The last variant was labeled as "Likely FP" because it was not detected by Sanger (it was noted as being possibly detected at very low levels) and found in only 1 of the 3 PacBio HiFi pipelines. While we left it as a FP, we are not confident in that decision because of the ambiguity in both the Sanger trace and the PacBio genotype calls. The degree of variability in evidence for the "Likely TP" and "Likely FP" variants may be further evidence that these regions are not yet "resolved" from a truth set perspective, and may have been incorrectly included in the benchmark files.

[3] HG006: https://ftp-trace.ncbi.nlm.nih.gov/ReferenceSamples/giab/data/ChineseTrio/HG006_NA24694-huCA017E_father/PacBio_CCS_15kb_20kb_chemistry2/reads/

[4] HG007: https://ftp-trace.ncbi.nlm.nih.gov/ReferenceSamples/giab/data/ChineseTrio/HG007_NA24695-hu38168_mother/PacBio_CCS_15kb_20kb_chemistry2/reads/

[5] minimap2: https://github.com/lh3/minimap2

[6] pbmm2: https://github.com/PacificBiosciences/pbmm2

[7] DeepVariant:https://github.com/google/deepvariant

Sample	Variant	Dragen Call	GIAB Call	HG006 “testing”	Sanger (GT)	PacBio HiFi (GT:DP)	Categorization and Other Notes
HG006	chr1:12760937C>T	0/1	0/0	0/1 - allfilteredbutagree	0/1	0/1:41	**Confirmed TP**; called 0/1 in 5/6 of the GIAB input supplement files
HG006	chr14:54613194A>G	0/1	0/0	0/1 - allfilteredbutagree	Primers failed	0/1:49	**Confirmed TP**; called 0/1 in 5/6 of the GIAB input supplement files
HG006	chr14:105926375G>A	0/1	0/0	0/0	0/0*	0/1:10 (1/3 pipelines)	**Likely FP**; visually detected in other PacBio BAMs, but was not called; called 0/1 in 4/6 of the GIAB input supplement files; *Sanger was noted as unclean, variant possibly detected at low levels
HG006	chr3:10927922C>A	0/1	0/0	0/1 - allfilteredbutagree	Primers failed	0/1:39	**Confirmed TP**; called 0/1 in 5/6 of the GIAB input supplement files
HG006	chr7:102283115G>A	0/1	0/0	0/1 - allfilteredbutagree	0/1	0/1:25	**Confirmed TP**; called 0/1 in 5/6 of the GIAB input supplement files
HG006	chr19:7116767TG>T	0/1	0/0	0/0	Primers failed	0/1:40	**Confirmed TP**; called 0/1 in 4/6 of the GIAB input supplement files
HG007	chr16:68157911A>G	0/1	0/0	N/A	0/1	0/0	**Likely TP**; missing from all GIAB input supplement files
HG007	chr11:56700783C>T	1/1	0/1	N/A	Primers failed	1/1:23	**Likely TP**; called 0/1 in all GIAB input supplement files

Table 17: Uncaptured FP Variant Analysis. This table contains summary results of our investigation of 8 variants which was labeled as false positives that were not captured using our trained models. We report the sample (HG006 or HG007), variant, the Dragen call, and the GIAB benchmark call on the left. On the right, we first have variants extracted from an HG006 “testing” file that can be found in the supplements along with any corresponding flags for that variant. The “allfilteredbutagree” flag has the description “All callsets have this call filtered or outside the callable regions but they have the same genotype.” Then, the genotype call (GT) results from Sanger sequencing for variants without primer failure are present. We also have the genotype call (GT) and depth of coverage (DP) for the PacBio HiFi sequencing. Finally, we categorized the variants and provided additional notes from our investigation in the final column. The 7 variants labeled as “Confirmed TP” or “Likely TP” were removed from our subsequent analyses.

6.5 Final Performance on HG006 and HG007

After removing incorrectly labeled false positive calls (see previous section), we were left with 1 uncaptured false positive call. Table 18 contains a summary of these results from both HG006 and HG007 exonic, benchmark regions. Overall, even with the one questionable false positive, the capture rate is still 99.70% with a TP Flag Rate of 12.99%. The TP flag rates in these exonic regions tends to be lower than the rates from our training and testing process, especially for indels. This suggests that the variants being called in exonic regions are perhaps "cleaner" than those elsewhere in the genome.

**Variant Type**	**Capture Rate (%)**	**TP Flag Rate(%)**
SNV - Heterozygous	99.35 (154/155)	10.58 (11365/107387)
SNV - Homozygous	100.0 (31/31)	12.43 (10193/81380)
SNV - Complex Het.	– (0/0)	100.00 (44/44)
Indel - Heterozygous	100.00 (116/116)	26.19 (2937/11216)
Indel - Homozygous	100.00 (23/23)	32.70 (2225/6805)
Indel - Complex Het.	100.00 (7/7)	31.56 (160/507)
**All Variants**	**99.70 (331/332)**	**12.99 (26924/207339)**

Table 18: HG006 and HG007 Experiment Results. This table shows the combined performance of our trained models for exonic benchmark variants from HG006 and HG007. Each variant type has its own model, and the performance in terms of false positive capture rate and TP flag rate is shown. Additionally, a combined summary is at the bottom labeled as "All Variants". Overall, only 1 false positive call was not captured while only 12.99% of true positives were flagged for confirmation.

7 Notes on application outside benchmark regions

From our prospective analysis, 88 of the 306 variants were not contained by any of the GIAB benchmark regions. Of these, four were primary or actionable, and five were non-actionable secondary findings. The remaining 79 were non-actionable, pharmacogenomic variants, most of which were from one of five genes: CYP2B6 (12), CYP2D6 (23), CYP4F2 (12), IFNL3 (16), and VKORC1 (12). Our lab has extensive experience reporting variants from these genes, and we were comfortable accepting the model limitations in this specific non-primary, non-actionable context. We recommend clinical labs initially apply the lowest risk approach (only non-actionable variant calls inside GIAB benchmark regions) and only consider other approaches after careful evaluation. We wish to reiterate that we do not currently recommend clinical labs apply these models to primary or actionable variants due to the increased risk to the patient. Furthermore, labs should be cautious even when applying the models to non-primary, non-actionable variants outside of the benchmark regions until further studies can be conducted to analyze their accuracy outside of GIAB benchmark regions.
